# Metformin attenuated sepsis-related liver injury by modulating gut microbiota

**DOI:** 10.1080/22221751.2022.2045876

**Published:** 2022-03-15

**Authors:** Huoyan Liang, Heng Song, Xiaojuan Zhang, Gaofei Song, Yuze Wang, Xianfei Ding, Xiaoguang Duan, Lifeng Li, Tongwen Sun, Quancheng Kan

**Affiliations:** a General ICU, The First Affiliated Hospital of Zhengzhou University, Henan Key Laboratory of Critical Care Medicine, Zhengzhou Key Laboratory of Sepsis, Henan Engineering Research Center for Critical Care Medicine, Zhengzhou, People’s Republic of China; b Academy of Medical Sciences, Zhengzhou University, Zhengzhou, People’s Republic of China; c Department of Pharmacy, The First Affiliated Hospital of Zhengzhou University, Internet Medical and System Applications of National Engineering Laboratory, Zhengzhou, People’s Republic of China; d Department of Pharmacy, The First Affiliated Hospital of Zhengzhou University, Zhengzhou, People’s Republic of China

**Keywords:** Sepsis-related liver injury, aged rats, metformin, gut microbiota, CLP

## Abstract

Increased evidence shows that gut microbiota acts as the primary regulator of the liver; however, its role in sepsis-related liver injury (SLI) in the elderly is unclear. This study assessed whether metformin could attenuate SLI by modulating gut microbiota in septic-aged rats. Cecal ligation and puncture (CLP) was used to induce SLI in aged rats. Fecal microbiota transplantation (FMT) was used to validate the roles of gut microbiota in these pathologies. The composition of gut microbiota was analysed by 16S rRNA sequencing. Moreover, the liver and colon tissues were analysed by histopathology, immunofluorescence, immunohistochemistry, and reverse transcription polymerase chain reaction (RT–PCR). Metformin improved liver damage, colon barrier dysfunction in aged SLI rats. Moreover, metformin improved sepsis-induced liver inflammation and damage under gut microbiota. Importantly, FMT assay showed that rats gavaged with faeces from metformin-treated SLI rats displayed less severe liver damage and colon barrier dysfunctions than those gavaged with faeces from SLI rats. The gut microbiota composition among the sham-operated, CLP-operated and metformin-treated SLI rats was different. In particular, the proportion of *Klebsiella* and *Escherichia_Shigella* was higher in SLI rats than sham-operated and metformin-treated SLI rats; while metformin could increase the proportion of *Bifidobacterium*, *Muribaculaceae*, *Parabacteroides_distasonis* and *Alloprevitella* in aged SLI rats. Additionally, *Klebsiella* and *Escherichia_Shigella* correlated positively with the inflammatory factors in the liver. Our findings suggest that metformin may improve liver injury by regulating the gut microbiota and alleviating colon barrier dysfunction in septic-aged rats, which may be an effective therapy for SLI.

## Introduction

Sepsis is characterised by overwhelming inflammation and aberrant immune responses, resulting in life-threatening organ dysfunction, high morbidity, and high mortality [1]. More than 1.5 million patients are subjected to sepsis annually, leading to about 33% mortality in the USA [2]. Sepsis activates the host’s immune responses, subsequently causing inflammatory storms and aberrant immune responses [3]. The cytokine storm further induces cell dysfunction and apoptosis, causing organ dysfunction and death. As the liver is a vital organ to balance the host homeostasis and regulate the host-defense effect, and the gut-liver axis become the key “target” organ of gut microbiota in sepsis. Sepsis-related liver injury (SLI) is associated with higher mortality [4], and a poor prognosis in SLI suggests a lack of clinically feasible therapeutic methods. Therefore, researching the pathogenesis of SLI to find a new effective therapy method is necessary.

Gut microbiota is the chief regulator of immune activities in the gut and extra-gut organs [5], and the liver is a vital organ that regulates the host’s metabolic and immune activities. Studies have reported that gut microbiota in the gastrointestinal tract remotely modulates multiple organ injuries, especially in the liver [6,7] and mediates the susceptibility to SLI [8]. Gut microbiota dysbiosis increases the risk of sepsis and death [9], while germ-free mouse models of sepsis showed a high pathogen burden and death rate [10]. Gut microbiota regulates the upstream of immune activities [5] and is involved in sepsis progression [8]. Therefore, gut microbiota may be a significant conductor of SLI pathogenesis.

Gut microbial composition varies with age [11], undergoing dramatic changes from infant to adult and from dominant *Bifidobacterium* to *Bacteroidetes* and *Clostridia* genus [11,12]. The composition change suggests a shift from catabolism to vitamin metabolism and short-chain fatty acid (SCFA) production, which inhibits intestinal inflammation but maintains mucosa and gut permeability. The gut microbial communities vary in adults and aged individuals [13]. Centenarians’ gut microbiota showed decreased *Clostridia*, *Eubacteriaceae*, *Faecalibacterium*, and *Lactobacillus* and increased *Proteobacteria* and *Bacilli*, suggesting that productive SCFA-related gut microbiota decreases with age, while pathogenic microbiota increases with age [14]. The data, therefore, indicate that changes in gut microbiota lead to increased inflammation and gut permeability. A previous study suggested that aged individuals have chronic low-grade inflammation that causes an aberrant increase in gut permeability [15]. Hence, maintaining gut microbiota homeostasis and reducing gut permeability may be a potential therapeutic method to decrease age-associated SLI.

Metformin, initially known as the first-line anti-diabetes agent, is beneficial in treating age-associated disorders and increasing the life span of animals [16, 17], highlighting its protective role in biologically aged mice and age-related mortality [17]. The wide use of metformin is safe and efficient in increasing insulin and insulin-related factors, inhibiting mitochondrial function, improving the metabolic and cellular processes associated with the development of age-related diseases (e.g. autophagy) [18] and cellular senescence [19]. One recent study [20] reported that metformin improves SLI by reducing the expression of hepatic plasminogen activator inhibitor type-1 and myeloperoxidase (MPO) activity and restoring the activity of antioxidants, such as glutathione. Metformin becomes concentrated in the gut due to oral administration [21], changing the dominant gut microbial composition and microbiota [22]. A double-blind study on the transfer of faecal samples from metformin-administered mice to germ-free mice confirmed the improved effect of metformin on gut microbiota due to altered metalloproteins or metal transporter homeostasis [23]. This study investigated the role of metformin in attenuating SLI by modulating gut microbiota in septic-aged rats to find an effective SLI therapy.

## Results

### Metformin attenuated cecal ligation and puncture (CLP) induced liver inflammation and injury in septic-aged rats

**Figure 1.**
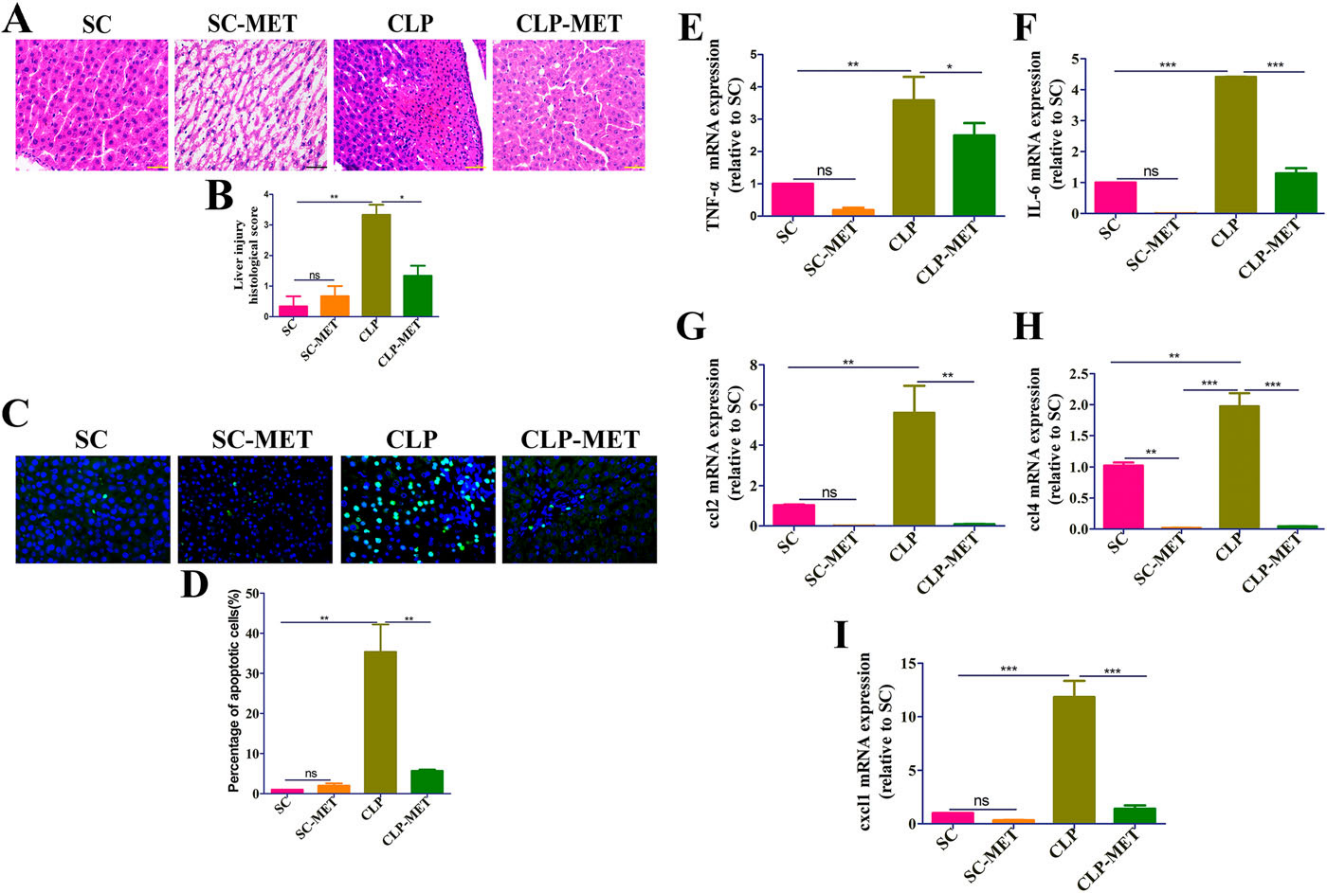
Metformin attenuated CLP-induced liver inflammation and injury in aged rats. (A,B) H&E staining showed significantly increased inflammatory infiltration, oedema, and haemorrhage of liver tissues in the CLP group than SC and SC-MET groups, whereas these abnormalities were markedly reduced in the CLP-MET group (bar = 50 μm; n = 3/group). (C,D) TUNEL assay results in four groups showed that the hepatocyte apoptosis increased in the CLP group and decreased in the CLP-MET group (n = 3/group). (E-I) The RT-PCR experiment showed that compared with SC and SC-MET groups, the expression of inflammatory factors TNF-α, IL-6, and chemokines ccl2, cxcl1, and ccl4 were elevated in the CLP group and decreased in the CLP-MET group. *P < 0.05, **P < 0.01, ***P < 0.001 (n = 3/group).

An earlier study [24] reported that increased body temperature is associated with lower mortality in adult septic patients, whereas the mortality of aged patients is not associated with body temperature. We observed in this study lower body temperature in the CLP group than the sham-operated (SC group), and metformin could reverse the sepsis-induced lower body temperature (*P *< 0.05; Supplementary Figure 1), while the 24-h survival analysis shows no difference between different groups (Supplementary Figure 2). Metformin induces kidney failure by lactic acidosis in the previous study [25], and it may be exacerbated in sepsis. Therefore, we performed a histological analysis on kidney tissues to check the kidney’s damage. The haematoxylin and eosin (H&E) and TUNEL assay results showed that the kidney tissue was not injured after the metformin administration (Supplementary Figure 3), suggesting that the current dose of metformin is safe and effective. To test liver inflammation and injury elicited by CLP-induced sepsis in aged rats, we performed the histological and reverse transcription-polymerase chain reaction (RT–PCR) analysis in the liver 24 h after CLP. The H&E examination of liver sections showed hepatic lobule destruction, inflammatory infiltration, and liver haemorrhage in the CLP group compared with SC and metformin treated-sham operated (SC-MET) groups. Metformin treatment could reverse the CLP-induced hepatic lobule destruction, inflammatory infiltration, and liver haemorrhage (*P *< 0.05; Figure 1(A,B)). Afterwards, we examined whether metformin could improve the sepsis-induced hepatocyte apoptosis of liver tissues in aged rats. The TUNEL assay showed that the hepatocyte apoptosis increased significantly in the CLP group compared with SC and SC-MET groups, whereas metformin treatment substantially reduced the sepsis-induced hepatocyte apoptosis (*P *< 0.05; Figure 1(C,D)). To confirm the effect of metformin on inflammatory factors and chemokines in the liver, we measured the mRNA expression of tumour necrosis factor-alpha (TNF-α), chemokine [C–C motif] ligand 2 (ccl2), interleukin-6 (IL-6), chemokine [C-X-C motif] ligand 1 (cxcl1), and ccl4. We also analysed serum biochemical parameters, such as alanine aminotransferase (ALT), aspartate aminotransferase (AST) in the liver of aged rats. The results showed that the gene expression of inflammatory factors TNF-α and IL-6, chemokines ccl2, cxcl1, and ccl4, as well as ALT and AST, increased markedly in the CLP group, whereas the increased levels of inflammatory factors and chemokines induced by sepsis were reversed in the metformin-treated-CLP (CLP-MET) group (*P *< 0.05; Figure 1(E–I), Supplementary Figure 4A-B). Taken together, these data indicated that metformin could attenuate inflammation and liver injury in septic-aged rats.

### The effect of metformin on inflammation and intestinal barrier of the colon in septic aged rats

**Figure 2.**
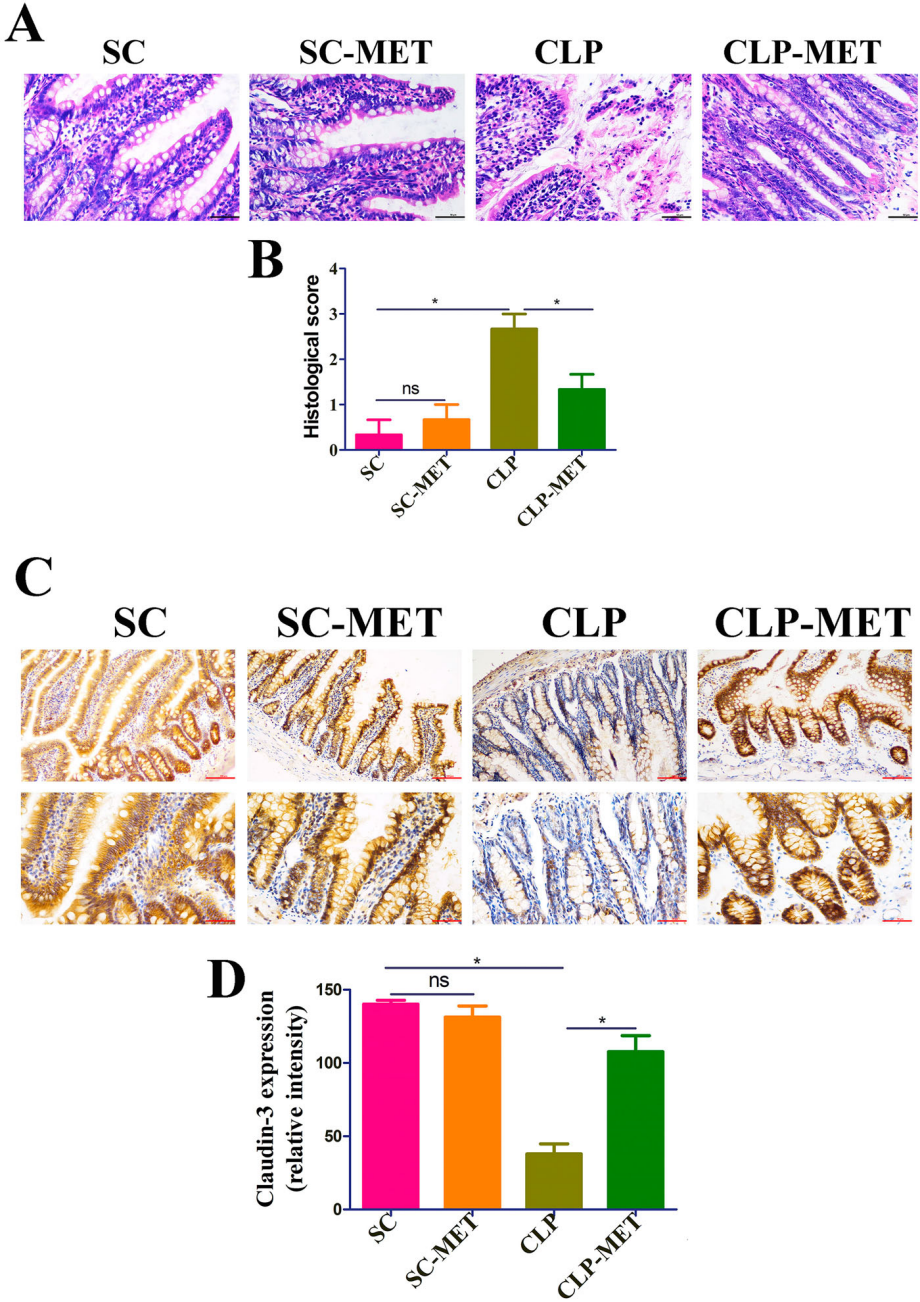
The effect of metformin on inflammation and intestinal barrier of the colon in aged rats. (A,B) H&E staining indicated that inflammatory infiltration, oedema, and haemorrhage of colon tissues significantly increased in the CLP group compared with SC and SC-MET groups, whereas these abnormalities improved in the CLP-MET group (bar = 100 μm). (C,D) The IHS expression results of claudin-3 protein expression (bar = 50/100 μm). *P < 0.05, **P < 0.01, ***P < 0.001 (n = 3/group).

Further examination of colon sections showed colon inflammation characterized by the eroded colonic mucosa, disorderly and deformed glands, fuzzy structures of tight junctions, and wide infiltration of inflammatory cells with crypt abscess in the CLP group. However, the CLP-MET group showed that rats had intact colonic epithelium, normal glands, and lesser inflammatory cell infiltration than in the CLP group (Figure 2(A,B)). These data suggested that CLP-induced sepsis could severely damage the colonic mucosa, and metformin treatment could significantly reduce this damage. As a leaky gut is usually found in aged adults [26], we performed the immunohistochemistry analysis to assess the expression of tight-junction proteins, claudin-3, in colon tissues. The expression of claudin-3 decreased in the CLP group, while metformin administration increased the expression of claudin-3 (Figure 2(C,D)). Collectively, these findings indicated that metformin treatment could increase the expression of tight-junction proteins, decreasing leaky gut and inflammation in aged SLI rats.

### The effect of metformin on SLI was transmissible by affecting the susceptibility of gut microbiota

**Figure 3.**
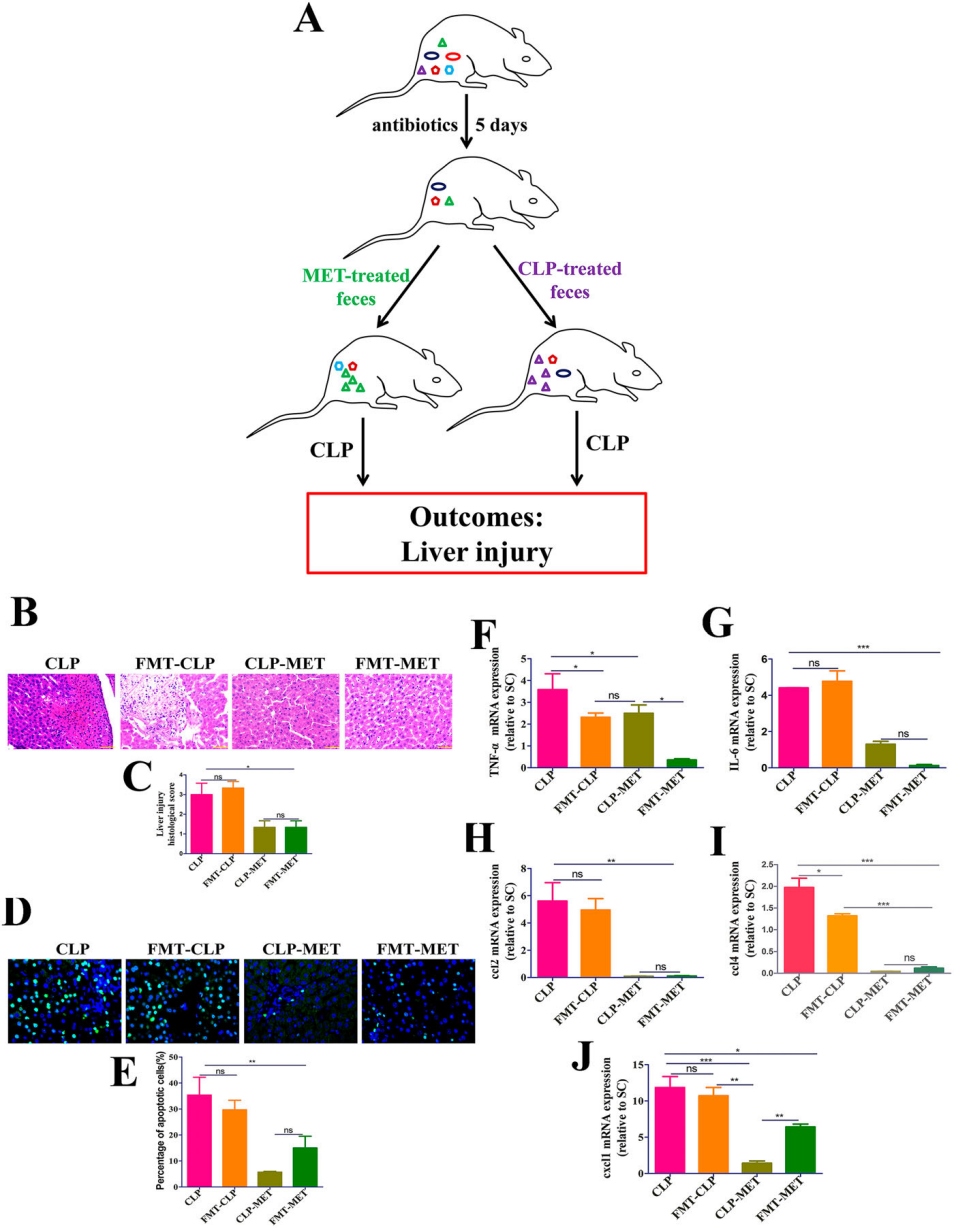
The effect of metformin on SLI was transmissible by affecting the susceptibility of gut microbiota. (A) FMT experimental design: Twelve rats received antibiotics (vancomycin, 100 mg/kg; neomycin sulphate, 200 mg/kg; metronidazole, 200 mg/kg; and ampicillin, 200 mg/kg) intragastrically once a day for 5 days to deplete the gut microbiota, producing Twelve pseudo-germ-free aged rats (recipient rats). Among them, 6 were transplanted with the CLP-treated faeces (n = 10) and 6 were transplanted with CLP-MET-treated feces (n = 10) once a day for 3 days. Thereby, FMT-CLP (n = 6) and FMT-MET groups (n = 6) were obtained, respectively. Faeces from donor rats (10 rats in CLP and CLP-MET groups, respectively) were collected and resuspended in phosphate buffer saline (PBS) at 0.125 g/mL, then 0.15 mL of this suspension was administered to rats by oral gavage once a day for 3 days. After 3 days, rats were subjected to CLP and sacrificed 24 h later, at which time tissues were collected. (B,C) H&E staining and the histological score of the liver from donors and sacrificed rats. (D,E) Hepatic TUNEL assay and quantification of donors and sacrificed rats. (F-J) The RT-PCR experiment showed that compared with CLP-MET and FMT-MET groups, the expression of inflammatory factors TNF-α, IL-6, and chemokines ccl2, cxcl1, and ccl4 were elevated in CLP and FMT-CLP groups. *P < 0.05, **P < 0.01, ***P < 0.001 (n = 3/group).

**Figure 4.**
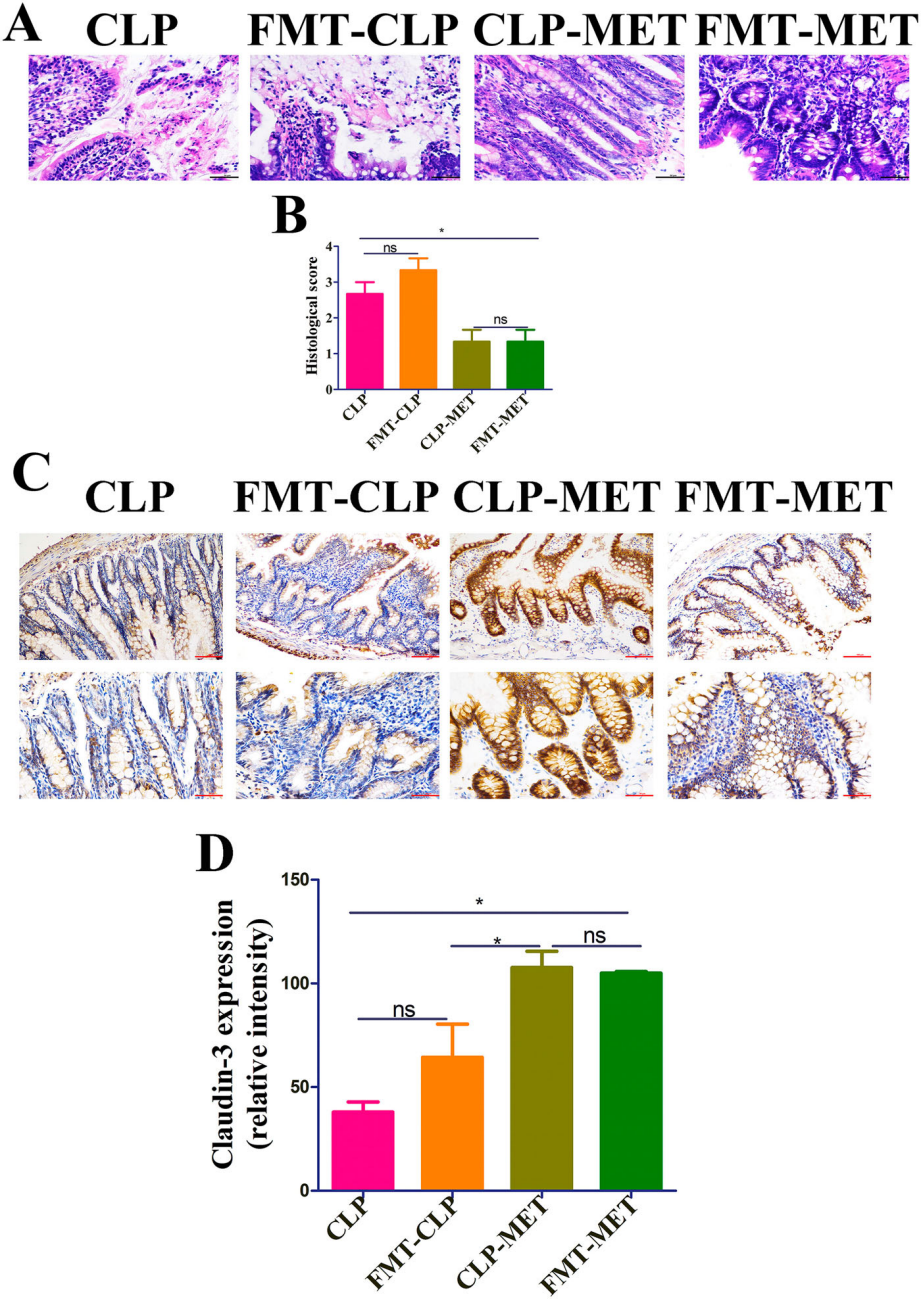
The effect of metformin on inflammation and intestinal barrier of the colon in the FMT experiment. (A,B) H&E staining indicated that inflammatory infiltration, oedema, and haemorrhage of colon tissues significantly increased in CLP and FMT-CLP groups compared with CLP-MET and FMT-MET groups (bar = 50 μm). (C,D) The IHS expression results of claudin-3 protein expression (bar = 50/100 μm). *P < 0.05, **P < 0.01, ***P < 0.001 (n = 3/group).

Subsequently, we searched whether metformin could mediate the difference in susceptibility of septic-aged rats. As the gut microbiota may be a vital modulator of the host response to polymicrobial sepsis [8], we speculated that metformin ameliorates SLI by mediating the gut microbiota. To verify our hypothesis, we first detected the effect of metformin on liver injury in the presence or absence of gut microbiota. The H&E examination of liver sections showed hepatic lobule destruction, inflammatory infiltration, and haemorrhage of the liver in metformin-treated septic-aged rats in the CLP-MET group compared with the Ab-CLP-MET group (*P *< 0.05; Supplementary Figure 5A-B). Furthermore, the TUNEL assay showed that the hepatocyte apoptosis increased significantly in metformin-treated septic-aged rats in the CLP-MET group compared with the Ab-CLP-MET group (*P *< 0.05; Supplementary Figure 5C-D). The mRNA expression of inflammatory factors TNF-α and IL-6, cxcl1, and ccl4 as well as ALT and AST increased markedly, while ccl2 decreased significantly in metformin-treated septic aged rats in the CLP-MET group compared with the Ab-CLP-MET group (*P *< 0.05; Supplementary Figure 5E-I, Supplementary Figure 4E-F). Then, we conducted a faecal microbiota transplantation (FMT) experiment (Figure 3(A)). Rats that received metformin-treated faeces developed less severe liver injury than that received CLP-treated faeces after CLP-induced sepsis, according to histological analysis and injury score quantification (Figure 3(B,C)). Furthermore, we also observed that the gene expression of inflammatory factors (TNF-α and IL-6) and chemokines (ccl2, cxcl1, and ccl4), as well as ALT and AST, decreased markedly in CLP-MET and metformin treated-FMT (FMT-MET) groups than in CLP and CLP treated-FMT (FMT-CLP) groups (*P *< 0.05; Figure 3(F–J), Supplementary Figure 4C-D). More importantly, the TUNEL assay showed that rats transplanted with CLP-MET-treated faeces after CLP exhibited fewer apoptotic cells compared with that received CLP-treated faeces (Figure 3(D,E)). More importantly, compared with the FMT-CLP group, the FMT-MET group showed improved eroded colonic mucosas, disorderly and deformed glands, fuzzy structures of tight junctions, and improved infiltrated inflammatory cells (Figure 4(A,B)). The expression of tight-junction proteins, claudin-3, was significantly increased in the colon tissue of the FMT-MET group (Figure 4(C,D)). Collectively, these data revealed that the effect of metformin on SLI was transmissible by affecting the susceptibility of gut microbiota.

### The beneficial effect of metformin on gut microbiota in aged SLI rats

**Figure 5.**
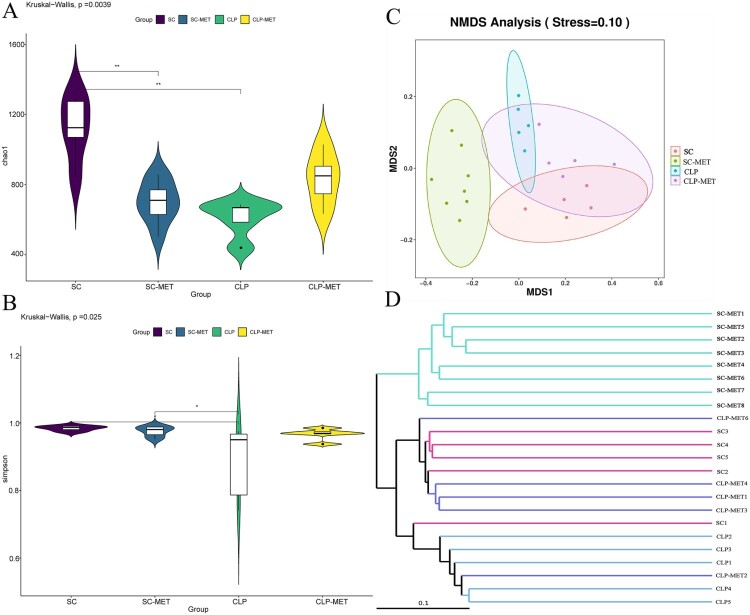
The effect of metformin on the diversity of gut microbiota in aged rats. (A,B) 16S rRNA gene sequencing analysis showed that the alpha diversity of gut microbiota, such as Chao1 and Shannon indices, was dramatically reduced in the CLP group compared with the SC group, whereas the alpha diversity was reversed in the CLP-MET group. (C,D) Beta diversity of gut microbiota, whose unweighted UniFrac NMDS analysis showed that the composition of the CLP group was different from that of SC and SC-MET groups. However, the composition of gut microbiota in the CLP-MET group was similar to the SC group (n = 5/group).

**Figure 6.**
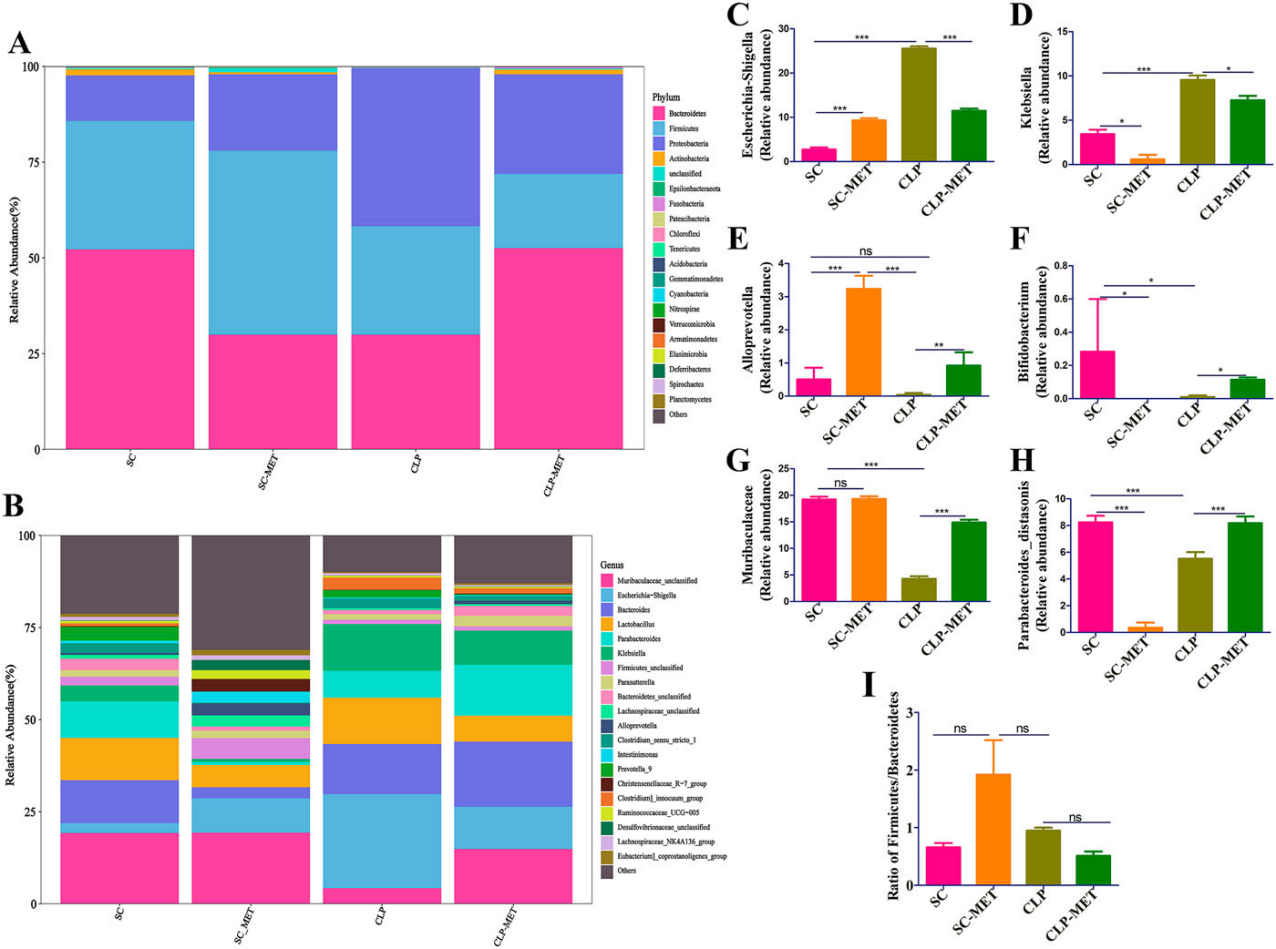
The beneficial effect of metformin on gut microbiota in aged rats. (A) The gut microbiota showed differences among SC, SC-MET, CLP, and CLP-MET groups in the phylum level. The relative abundance of *Bacteroidetes* decreased in CLP and SC-MET groups compared with the SC group, but it was reversed in the CLP-MET group. (B) The Genus-level gut microbiota showed differences among SC, SC-MET, CLP, and CLP-MET groups. (C-H) The abundance of *Escherichia_Shigella*, *Klebsiella* increased in the CLP group compared with SC and SC-MET groups, but it was reversed in the CLP-MET group; the relative abundance of *Bifidobacterium*, *Muribaculaceae*, *Parabacteroides_distasonis*, and *Alloprevitella* decreased in the CLP group compared with SC and SC-MET groups, and it was increased in the CLP-MET group. (I) The ratio of *Firmicutes/Bacteroidetes* slightly increased in SC-MET and CLP groups compared with the SC group. However, metformin treatment could slightly decrease the ratio of *Firmicutes/Bacteroidetes* compared with the CLP group. *P < 0.05, **P < 0.01, ***P < 0.001.

To determine the impact of SLI and metformin interactions on the gut microbiota of aged SAL rats, a gut analysis was performed. The results indicated that the alpha diversity of gut microbiota in terms of alpha diversity indices (Chao1 and Shannon) was dramatically reduced in the CLP group than the SC group. Surprisingly, the alpha diversity indices also decreased in the SC-MET group. However, metformin treatment restored the alpha diversity reduction caused by CLP-induced sepsis slightly (Figure 5(A,B)), suggesting that metformin could improve the alpha diversity of gut microbiota induced by sepsis. We further examined the beta diversity (variation in gut microbial composition among groups) of gut microbiota among groups. Intriguingly, the gut microbial composition of the SC-MET group was different from the other three groups; and gut microbial composition of the CLP-MET group was more similar to the SC group than the CLP group (Figure 5(C,D)). The gut microbiota showed differences among SC, SC-MET, CLP, and CLP-MET groups in the phylum level. The relative abundance of *Bacteroidetes* decreased in CLP and SC-MET groups compared with the SC group, but it was reversed in the CLP-MET group; and the ratio of *Firmicutes/Bacteroidetes* slightly increased in SC-MET and CLP groups compared with the SC group. However, metformin treatment slightly increased the ratio of *Firmicutes/Bacteroidetes* compared with the CLP group (Figure 6(A,I)). Importantly, the relative abundance of *Escherichia_Shigella* and *Alloprevotel* increased, while *Klebsiella*, *Bifidobacterium*, and *Parabacteroides_distasonis* decreased in the SC-MET group. Additionally, the relative abundance of *Alloprevotella*, *Bifidobacterium*, *Parabacteroides_distasonis*, and *Muribaculaceae* related to the SCFA production and anti-inflammation decreased, whereas that of *Escherichia_Shigella* and *Klebsiella* related to the lipopolysaccharide (LPS) production markedly increased in the gut microbiota of the CLP group compared with the SC group. Conversely, the ratio of *Escherichia_Shigella* and *Klebsiella* decreased, and that of *Alloprevotella*, *Alphaproteobacteria*, *Bifidobacterium, Parabacteroides_distasonis* and *Muribaculaceae* increased in the CLP-MET group compared with the CLP group (*P *< 0.05; Figure 6(B–H)). These results highlighted the importance of the regulatory effect of metformin on gut microbiota, especially on SLI.

**Figure 7.**
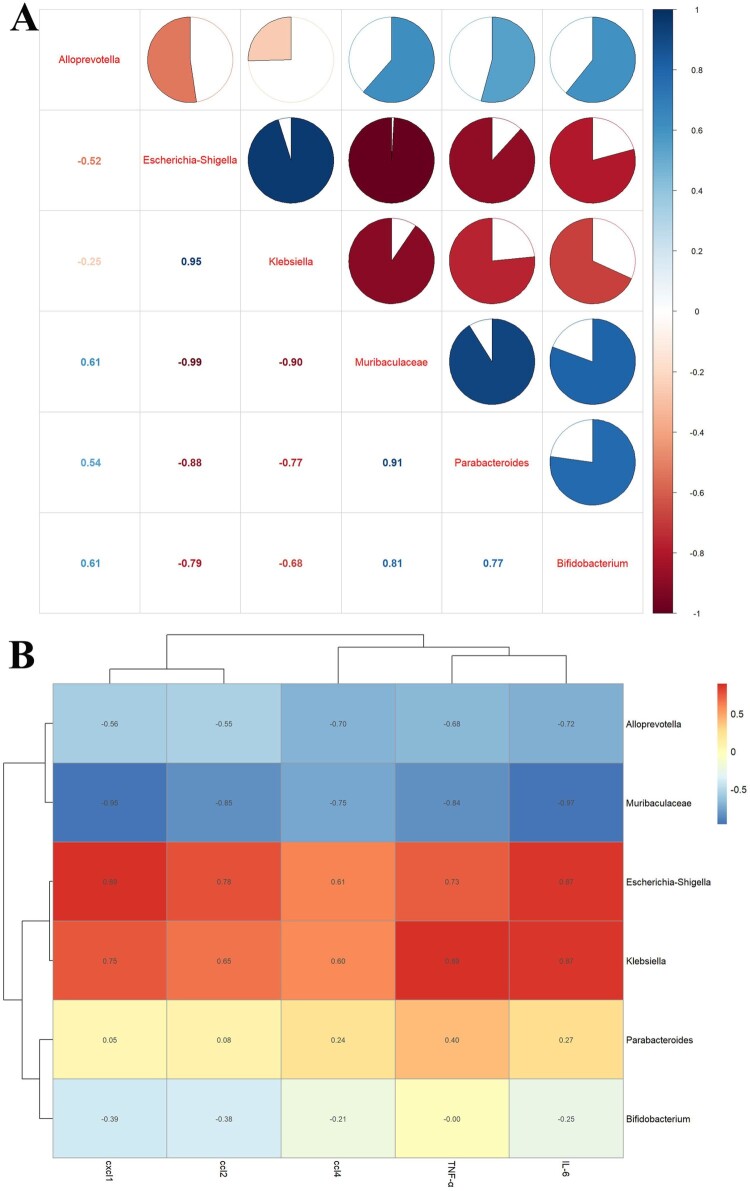
The correlation analysis of the gut microbiota and liver inflammation in aged rats. (A) The correlation analysis of the gut microbiota showed that *Escherichia_Shigella*, *Klebsiella* were negatively correlated with *Alloprevitella*, *Bifidobacterium*, *Muribaculaceae*, and *Parabacteroides_distasonis*; *Muribaculaceae* was positively correlated with *Parabacteroides_distasonis* and *Bifidobacterium*. (B) The correlation analysis between gut microbiota and liver inflammation showed that TNF-α, IL-6, ccl2, cxcl1, and ccl4 of the liver showed a positive correlation with *Escherichia_Shigella*, *Klebsiella*, and a negative correlation with *Muribaculaceae* and *Alloprevitella*.

Strikingly, the correlation analysis of gut microbiota among groups showed that *Bifidobacterium, Parabacteroides_distasonis* and *Muribaculaceae* were negatively correlated with *Klebsiella* and *Escherichia_Shigella*, and *Muribaculaceae* was positively correlated with *Alloprevotella* (Figure 7(A)). We researched the correlation between gut microbiota and inflammatory factors, and the results showed that *Klebsiella* and *Escherichia_Shigella* were positively correlated with TNF-α, IL-6, ccl2, cxcl1, and ccl4 (Figure 7(B)). The Phylogenetic Investigation of Communities by Reconstruction of Unobserved States (PICRUSts) analysed the differences in the Kyoto Encyclopedia of Genes and Genomes (KEGG) metabolic pathway among groups. In particular, the gut microbiota metabolism may be enriched in some pathways. For example, glucose metabolism, vitamin metabolism, amino acid metabolism, and polyisoprenoid metabolism were significantly enhanced in the SC faeces compared with those in the SC-MET feces (Supplementary Figure 6A); glucose metabolism, vitamin metabolism, and polyisoprenoid metabolism were slightly enhanced in SC faeces compared with those in the CLP feces (Supplementary Figure 6B); vitamin metabolism was significantly enhanced in the CLP-MET faeces compared with that in the CLP faeces (Supplementary Figure 6C). Collectively, these data suggested that metformin may affect the metabolic pathways of gut microbiota in aged SLI rats.

## Discussion

This study researched the role of metformin in improving inflammation and liver injury by modulating the gut microbiota in septic-aged rats. The composition of gut microbiota in the CLP-MET group changed from LPS production and inflammation to SCFA production and anti-inflammation, which protected rats from SLI. The gut microbiota shifted to SCFA production after metformin treatment, which may increase the claudin-3 level in the colon, suppressing the leaky gut and protecting it from cytokine inflammation production. Our work supports the general idea of using the FMT assay in sepsis because metformin protects inflammatory responses and liver injury in sepsis.

Metformin is the first-line therapy for treating type 2 diabetes mellitus (T2DM), and subsequently, multiple other therapeutic efficacies have been researched. Particularly, the effect of metformin on various diseases by changing the gut microbiota has aroused widespread concerns. The previous study [16] reported that metformin retards ageing in C. elegans by altering microbial folate and methionine metabolism, which provides an effective treatment for ageing prevention. A subsequent study [23] suggested that metformin exerts an anti-diabetic effect by regulating the microbial encoded metalloproteins or metal transporters, and a recent article [27] elucidated that metformin can act as a regulator to affect the host lipid metabolism and life span. More and more evidence suggests that microbiome changes on metformin administration were previously described and linked to some host effects of this drug. It is believed that metformin affects SLI by modulating the microbiome at least in part. However, some studies [23,28–31] reported that an increase in the abundance of *Escherichia-Shigella* in metformin treatment was observed in healthy volunteers and patients with T2DM or other diseases. Our results showed the relative abundance of *Escherichia-Shigella* increased in the SC-MET group compared with the SC group, and the ratio of *Firmicutes/Bacteriodetes* slightly decreased in the CLP-MET group compared with the CLP group, which is consistent with the results of previous studies. Additionally, a report [29] showed that metformin treatment creates a competitive environment for *Escherichia coli* using other energy sources, thereby inducing the abundance of gut microbiome alters [32]. The subsequent article [23] indicated the relative abundance of *Escherichia coli* can have an effect on metformin administration under the vitro gut simulation. A recent study [30] showed that the abundance of *Escherichia/Shigella* before metformin treatment is associated with side effects. Furthermore, a study implied that *Escherichia* is a marker of gastrointestinal side effects upon metformin administration [29], and the authors also clarified that side effects derived from *Escherichia* are caused by an increase in LPS synthesis or sulphate metabolism potential.

The FMT assay in this study highlighted the role of the gut microbiota as an upstream conductor of sepsis-induced inflammation and liver injury. More importantly, our results showed that metformin might improve SLI in aged rats in the presence of gut microbiota compared with the absence of gut microbiota. Furthermore, our observations that metformin treatment could improve inflammation and liver injury by regulating the gut microbiota and alleviating leaky gut in aged SLI rats were consistent with those of the previous study [33]. An earlier article reported that a leaky gut regulates inflammation, associated with a higher risk of metabolic dysfunction in aged individuals [34]. Our results were consistent with an earlier study, which reported that the reduced expression of intestinal barrier markers, such as tight-junction proteins, results in a leaky gut [35]. Moreover, CLP-induced colon inflammatory infiltration, oedema, and haemorrhage also promote the leaky gut. The bacteria, especially LPS, translocated to the extraintestinal through the impaired intestinal barrier, stimulate liver injury and inflammation [36]. It is consistent with our finds in the colon inflammatory infiltration, oedema, and haemorrhage responses to liver injury and inflammation.

This study showed that the gut microbial composition differed substantially between SC and CLP groups (Figure 5). Surprisingly, metformin treatment leads to a decreased alpha diversity (SC-MET group), which may be due to gastrointestinal side effects upon metformin administration. Furthermore, metformin administration could increase the abundance of *Escherichia_Shigella*. The increased levels of opportunistic pathogenic bacteria with a pro-inflammatory response, such as *Klebsiella* and *Escherichia_Shigella*, in CLP-induced sepsis destroy the gut microe-cological balance, while the activated intestinal pathogenic bacteria increase inflammation, which is in agreement with our previous findings [37]. Another study reported that *Escherichia_Shigella* is involved in obesity-associated metabolic dysfunction or considered a pro-inflammatory bacterial species [38]. Our study also showed that *Klebsiella* and *Escherichia_Shigella* were positively correlated with liver inflammation and chemokines. As expected, the gut microbial composition in the CLP-MET group was similar to the SC-MET group (Figure 5). The present study showed that metformin treatment increased the abundance of *Bifidobacterium*, *Muribaculaceae*, *Parabacteroides_distasonis*, and *Alloprevitella* in the CLP-MET group compared with the CLP group. These bacteria produce SCFA, which provides energy to intestinal cells, maintains the gut barrier [39], and prevents LPS translocation from the intestinal barrier [40, 41]. *Alloprevitella* protects from acute liver injury and gut microbiota dysbiosis [42]. Increasing evidence reported that *Parabacteroides_distasonis* alleviate tumourigenesis, inflammatory markers, and intestinal barrier integrity in mice [43]; the bacteria are negatively correlated with AST and ALT [44] and can attenuate inflammation by inhibiting Toll-like receptor 4 signalling [45]. *Bifidobacterium* and *Muribaculaceae* have beneficial effects on intestinal dysbiosis through immunoregulation and modulation of gut homeostasis [46,47].

There are some shortcomings in this study. First, the samples in each group are small, and the study results need to be confirmed by other similar studies. Furthermore, although we detected the effect of metformin on liver injury and inflammation in the presence or absence of gut microbiota in aged SLI rats, the metformin concentration in the faeces of donors was not detected before the FMT assay. It may affect the result reliability as the unprocessed metformin in the faeces samples may act directly on the recipient animals. More importantly, we did not detect the specific metabolites in this study, as metformin directly inhibited the host immune system [48,49]. Therefore, it cannot be ruled out that metformin may also play a part by inhibiting the immune function of the host, which is the key driver of septic organ damage. This may underlay the protective properties of metformin in sepsis.

## Conclusion

The results of our study provided convincing evidence that metformin improved leaky gut, inflammation, and liver injury by beneficially regulating the gut microbiota dysbiosis induced by sepsis. *Klebsiella* and *Escherichia_Shigella* were significantly elevated in aged SLI rats; metformin treatment could reverse the gut microbiota dysbiosis and increase the abundance of *Bifidobacterium*, *Muribaculaceae*, *Parabacteroides_distasonis*, and *Alloprevitella*. These findings suggested that metformin could be used for the sepsis-induced leaky gut therapy, colon inflammation, and liver injury accompanied by gut microbiota dysbiosis in septic-aged rats.

## Methods

### Experimental procedures

This study was conducted according to the National Institutes of Health guidelines and was approved by the local Animal Care and Use Committee of Zhengzhou University (Henan, China).

### Animal experiment

Sixty male Sprague–Dawley (SD) rats (6–8 weeks) were purchased from Beijing Vital River Laboratory Animal Technology (Beijing, China). All were kept in 2 rats, each cage in a specific pathogen-free (SPF) animal laboratory with a temperature-controlled colony and 12/12 h light/dark cycle with free access to chow and water, and 55 rats were left until 20–21 months. Fifty-five aged male SD rats were randomly divided into 7 groups: SC group (n = 5), sham operation; SC-MET group (n = 8), metformin (100 mg/kg, dissolved in saline) administration by gavaging 1 h after sham operation; CLP group (n = 10), CLP-induced SLI rat model; CLP-MET group (n = 10), metformin administration by gavaging 1 h after CLP administration; Ab-CLP-MET group (n = 10), the aged rats were gavaged with compound antibiotics (vancomycin, 100 mg/kg; neomycin sulphate, 200 mg/kg; metronidazole, 200 mg/kg; and ampicillin, 200 mg/kg) once a day for 5 days to deplete the gut microbiota (pseudo-germ free aged rats, namely, recipient rats), and then metformin administration was conducted by gavaging 1 h after CLP administration; the FMT-CLP group (n = 6), we collected the compound faeces of 10 rats in the CLP group and gave them to the 6 recipient rats; the FMT-MET group (n = 6), we collected the compound feces of 10 rats in the CLP-MET group and gave them to the other 6 recipient rats. All the rats were sacrificed at 24 h after the CLP or sham-operated, we collected the relevant samples (Supplementary Figure 7). The CLP-induced septic rate model was based on our previous description [37]. Briefly, the rats were anaesthetized by intraperitoneally injecting chloral hydrate (10%, 40 mg/kg). Half of the cecum-free end was ligated and punctured twice with a 21-gauge needle. The cecum contents were squeezed out, the cecum was repositioned, and an incision was sutured in muscle and skin layers. Subsequently, the rats were subcutaneously injected with saline (1 mL/100 g) at 37°C, and returned to the cage to rewarm for 1 h. The SC group had an abdominal incision, but their cecum was not ligated and punctured, and the SC-MET group was treated with metformin based on the SC group.

### FMT assay

FMT assay was conducted based on the modified method described [8,25,26]. Briefly, twelve rats received compound antibiotics (vancomycin, 100 mg/kg; neomycin sulphate, 200 mg/kg; metronidazole, 200 mg/kg; and ampicillin, 200 mg/kg) intragastrically once a day for 5 days to deplete the gut microbiota, producing twelve pseudo-germ-free aged rats (recipient rats). The compound faeces of 10 CLP group rats were transplanted into the 6 recipients by gavage (FMT-CLP, n = 6), and the compound faeces of 10 CLP-MET group rats were transplanted into the other 6 recipients by gavage(FMT-MET, n = 6) once a day for 3 days. Faeces from donor rats (10 rats in CLP and CLP-MET groups, respectively) were collected and resuspended in phosphate buffer saline (PBS) at 0.125 g/mL, then 0.15 mL of this suspension was administered to rats by oral gavage once a day for 3 days. After 3 days, rats were subjected to CLP and sacrificed 24 h later, at which time tissues were collected.

### Histological analysis

The liver, kidney tissue, and colon tissue were washed with sterile normal saline, fixed in 4% paraformaldehyde for 24 h, and embedded in paraffin for H&E staining. The pathological score was evaluated based on previous studies [10,50]. Briefly, the parameters for inflammation, thrombus formation, and necrosis were graded on a scale of 0–4, with 0 defined as “absent” and 4 defined as “severe.” The total “histological score of liver” was expressed as the sum of the scores for each parameter, with a maximum score of 12 [8]. Colon sections were scored on congestion, intestinal wall thickening, ulcer, adhesion, using the scale given above, with 0 defined as “absent” and 4 defined as “severe.” The tissues were sectioned at 3–5 µm and stained with H&E for light microscopic analysis. We quantified oedema, haemorrhage in colon and liver tissues. To better assess the colon and liver lesions, we chose three sections and three regions within each section; the end score for each animal was calculated as the mean score of each section.

The paraffin sections of liver and kidney samples were prepared as previously described [51]. The terminal deoxynucleotidyl transferase dUTP nick-end labelling TUNEL assay was used to label the DNA fragment of apoptotic cells (brown nuclear staining) using the In Situ Cell Death Detection Kit, POD (Roch, Germany) in the 2–4 μm thin liver tissue sections according to the manufacture’s instructions. The Nikon Imager 2 light microscope was used to take pictures. The Image J scoring system was adopted for semi-quantitative analyses of apoptosis cells in liver and kidney tissues. Furthermore, the paraffin sections of colon samples were used in the IHS analysis to evaluate the expression of claudin-3 (1:100, ab15102, Abcam, USA) in colon tissues to assess the colon barrier function.

### Biochemical analysis

We used the commercial kits (Jiancheng Bioengineering) to measure the serum expressions of ALT and AST activities based on the manufacturer’s instructions.

### Quantification of mRNAs by RT–PCR

Total RNA of the liver was extracted using TRIzol reagent (Takara, Tokyo, Japan) following the manufacturer’s instructions. The TaqMan Reverse Transcription Kit (UE, Suzhou, China) and a Gene Amp polymerase chain reaction (PCR) System generated cDNA. The PCR was performed with the qPCR superMIX (Yeasen, Shanghai, China). The results were analyzed by the 2^-ΔΔCT^ method. Gene expression data were shown as relative to the SC group, which was set as 100%. The reduced glyceraldehyde-phosphate dehydrogenase (GAPDH) expression was used for gene expression normalization. The genes primers were TNF-α forward primer: CGTCAGCCGATTTGCCATTT, reverse primer: CTCCCTCAGGGGTGTCCTTA; ccl2 forward primer: TAGCATCCACGTGCTGTCTC, reverse primer: CAGCCGACTCATTGGGATCA; IL-6 forward primer: AGAGACTTCCAGCCAGTTGC, reverse primer: AGTCTCCTCTCCGGACTTGT; ccl4 forward primer: CGTGTCTGCCTTCTCTCTCC, reverse primer: GCACAGATTTGCCTGCCTTT; cxcl1 forward primer: CGCTCGCTTCTCTGTGCA, reverse primer: TTCTGAACCATGGGGGCTTC; GAPDH forward primer: TGTGAACGGATTTGGCCGTA, reverse primer: GATGGTGATGGGTTTCCCGT.

### 16S rRNA gene sequencing

Total genomic DNA from cecum feces stored in −80°C was extracted using the cetyltrimethylammonium bromide/sodium dodecyl sulphonate (CTAB/SDS) method. DNA concentration and purity were monitored on 1% agarose gel. According to the concentration, DNA was diluted to 1 ng/μL using sterile water. 16S rRNA genes of distinct regions (16S V3-V4) were amplified using the specific primer (341F: CCTAYGGGRBGCASCAG; 806R: GGACTACNNGGGTATCTAAT) with the barcode. All PCR reactions were carried out with Phusion® High-Fidelity PCR Master Mix (New England Biolabs, Massachusetts, USA). Same volumes of 1X loading buffer (SYB green) were mixed with PCR products and subjected to electrophoresis on 2% agarose gel for detection. Samples with bright main strips between 400and 450 bp (16S) and ITS (100–400 bp) were chosen for further experiments. PCR products were mixed in equidensity ratios. Then, the mixture of PCR products was purified with Qiagen Gel Extraction Kit (Qiagen, Germany). Sequencing libraries were generated using TruSeq® DNA PCR-Free Sample Preparation Kit (Illumina, USA) following the manufacturer’s recommendations, and index codes were added. The library quality was assessed on the Qubit® 2.0 Fluorometer (ThermoScientific) and Agilent Bioanalyzer 2100 system. Finally, the library was sequenced on an Illumina NovaSeq 6000 platform, and 250 bp paired-end reads were generated. The raw tags were double-ended reads, which could be accessed through fastq join (v1.3.1) (https://code.google.com/p/ea-utils/) and pear (v0.9.11). The original sequence contained two primer sequences, and then Cutadapt (v1.18) was used to remove sequences without primers and cut out fully sequenced primers from the reads. Q30. Usearch (version 11.0.667) with no ambiguous bases was used to cluster according to 97% similarity. Alpha diversity (Chao1 and Shannon) was calculated using Mothur v1.42.1, beta diversity (non-metric multidimensional scaling (NMDS) and unweighted pair-group method with arithmetic means (UPGMA)) was calculated using the vegan package in R-package, and pathway enrichment was calculated using PICRUSt2 software package (https://github.com/picrust/picrust2) [52].

### Reagents

Metformin, chloral hydrate, vancomycin, neomycin sulphate, metronidazole, and ampicillin were purchased from MedChemExpress (Monmouth Junction, NJ, USA). In Situ Cell Death Detection Kit, POD was bought from Roch (Germany). AST and ALT kits were purchased from Jiancheng Bioengineering (AST, C010-2-1; ALT, C009-2). Anti-claudin-3 was bought from Abcam (1:100, ab15102, USA). TRIzol reagent was purchased from Takara (Tokyo, Japan). TaqMan Reverse Transcription Kit was bought from UE (Suzhou, China). qPCR superMIX was purchased from Yeasen (Shanghai, China).

### Statistical analysis

Statistical analyses were performed using R-package and GraphPad Prism version 5.0 (GraphPad Software, La Jolla, CA, USA) . Mean ± standard deviation assessed the quantitative data, and the variance analyzed the comparison among more than three groups (SC vs. SC-MET vs. CLP vs. CLP-MET; CLP vs. CLP-MET vs. FMT-CLP vs. FMT-MET). We used the t-test or two-way analysis of variance (ANOVA) and Bonferroni post-test instead of individual compared to statistically analyze the data. Alpha diversity was calculated using Mothur v1.42.1, beta diversity was calculated using permutational ANOVA to compare groups (vegan package in R-package), and pathway enrichment was calculated using PICRUSt2 software package (https://github.com/picrust/picrust2) [52]. Furthermore, Spearman’s rank correlation coefficients were calculated for correlation analysis, and the R-package Corrplot, ggcorplot and pheatmap were used for the correlation matrix visualization. All the results among the groups were analyzed using a statistical significance level set at *P *< 0.05.

## Supplementary Material

Supplemental Material

## Data Availability

The data that support the findings of this study are available from the corresponding author upon reasonable request.

## Abbreviations

Abbreviations: Ab: antibiotic; ALT: alanine aminotransferase; AST: aspartate aminotransferase; CLP: cecal ligation and puncture; FMT: faecal microbiota transplantation; H&E: haematoxylin and eosin; IL: interleukin; MET: metformin; PICRUSt: Phylogenetic Investigation of Communities by Reconstruction of Unobserved States. RT–PCR: reverse transcription polymerase chain reaction; SC: sham-operated; SCFA: short-chain fatty acid; SLI: sepsis-related liver injury; TNF-α: tumour necrosis factor-alpha; cxcl/ccl: chemokine [C–C motif] ligand.

## References

[1] DeMerle KM, Angus DC, Baillie JK, et al. Sepsis subclasses: a framework for development and interpretation. Crit Care Med. 2021;49(5):748–759. 33591001 10.1097/CCM.0000000000004842 PMC8627188

[2] Buchman TG, Simpson SQ, Sciarretta KL, et al. Sepsis among medicare beneficiaries: 1. The burdens of sepsis, 2012-2018. Crit Care Med. 2020;48(3):276–288. 32058366 10.1097/CCM.0000000000004224 PMC7017943

[3] Fajgenbaum DC, June CH. Cytokine storm. N Engl J Med. 2020;383(23):2255–2273. 33264547 10.1056/NEJMra2026131 PMC7727315

[4] Savio LEB, de Andrade Mello P, Figliuolo VR, et al. CD39 limits P2X7 receptor inflammatory signaling and attenuates sepsis-induced liver injury. J Hepatol. 2017;67(4):716–726. 28554875 10.1016/j.jhep.2017.05.021 PMC5875702

[5] Honda K, Littman DR. The microbiota in adaptive immune homeostasis and disease. Nature. 2016;535(7610):75–84. 27383982 10.1038/nature18848

[6] Li D, Ke Y, Zhan R, et al. Trimethylamine-N-oxide promotes brain aging and cognitive impairment in mice. Aging Cell. 2018;17(4):e12768. 29749694 10.1111/acel.12768 PMC6052480

[7] Sun J, Zhang J, Wang X, et al. Gut-liver crosstalk in sepsis-induced liver injury. Crit Care. 2020;24(1):614. 33076940 10.1186/s13054-020-03327-1 PMC7574296

[8] Gong S, Yan Z, Liu Z, et al. Intestinal microbiota mediates the susceptibility to polymicrobial sepsis-induced liver injury by granisetron generation in mice. Hepatology (Baltimore, MD). 2019;69(4):1751–1767. 10.1002/hep.30361 30506577

[9] Liu Z, Li N, Fang H, et al. Enteric dysbiosis is associated with sepsis in patients. FASEB J Off Publ Federation American Soc Exp Biol. 2019;33(11):12299–12310. 10.1096/fj.201900398RR PMC6902702 31465241

[10] Schuijt TJ, Lankelma JM, Scicluna BP, et al. The gut microbiota plays a protective role in the host defence against pneumococcal pneumonia. Gut. 2016;65(4):575–583. 26511795 10.1136/gutjnl-2015-309728 PMC4819612

[11] Yatsunenko T, Rey FE, Manary MJ, et al. Human gut microbiome viewed across age and geography. Nature. 2012;486(7402):222–227. 22699611 10.1038/nature11053 PMC3376388

[12] Mariat D, Firmesse O, Levenez F, et al. The firmicutes/Bacteroidetes ratio of the human microbiota changes with age. BMC Microbiol. 2009;9(123). 10.1186/1471-2180-9-123 PMC2702274 19508720

[13] Claesson MJ, Jeffery IB, Conde S, et al. Gut microbiota composition correlates with diet and health in the elderly. Nature. 2012;488(7410):178–184. 22797518 10.1038/nature11319

[14] Rampelli S, Candela M, Turroni S, et al. Functional metagenomic profiling of intestinal microbiome in extreme ageing. Aging. 2013;5(12):902–912. 24334635 10.18632/aging.100623 PMC3883706

[15] Buford TW, Carter CS, VanDerPol WJ, et al. Composition and richness of the serum microbiome differ by age and link to systemic inflammation. GeroScience. 2018;40(3):257–268. 29869736 10.1007/s11357-018-0026-y PMC6060185

[16] Cabreiro F, Au C, Leung K-Y, et al. Metformin retards aging in C. elegans by altering microbial folate and methionine metabolism. Cell. 2013;153(1):228–239. 23540700 10.1016/j.cell.2013.02.035 PMC3898468

[17] Barzilai N, Crandall JP, Kritchevsky SB, et al. Metformin as a tool to target aging. Cell Metab. 2016;23(6):1060–1065. 27304507 10.1016/j.cmet.2016.05.011 PMC5943638

[18] Song YM, Lee YH, Kim JW, et al. Metformin alleviates hepatosteatosis by restoring SIRT1-mediated autophagy induction via an AMP-activated protein kinase-independent pathway. Autophagy. 2015;11(1):46–59. 25484077 10.4161/15548627.2014.984271 PMC4502778

[19] Moiseeva O, Deschênes-Simard X, St-Germain E, et al. Metformin inhibits the senescence-associated secretory phenotype by interfering with IKK/NF-κB activation. Aging Cell. 2013;12(3):489–498. 23521863 10.1111/acel.12075

[20] Ismail Hassan F, Didari T, Khan F, et al. A review on the protective effects of metformin in sepsis-induced organ failure. Cell J. 2020;21(4):363–370. 31376317 10.22074/cellj.2020.6286 PMC6722446

[21] Buse JB, DeFronzo RA, Rosenstock J, et al. The primary glucose-lowering effect of metformin resides in the gut, not the circulation: results from short-term pharmacokinetic and 12-week dose-ranging studies. Diabetes Care. 2016;39(2):198–205. 26285584 10.2337/dc15-0488

[22] Whang A, Nagpal R, Yadav H. Bi-directional drug-microbiome interactions of anti-diabetics. EBioMedicine. 2019;39:591–602. 30553752 10.1016/j.ebiom.2018.11.046 PMC6354569

[23] Wu H, Esteve E, Tremaroli V, et al. Metformin alters the gut microbiome of individuals with treatment-naive type 2 diabetes, contributing to the therapeutic effects of the drug. Nat Med. 2017;23(7):850–858. 28530702 10.1038/nm.4345

[24] Inghammar M, Sunden-Cullberg J. Prognostic significance of body temperature in the emergency department vs the ICU in patients with severe sepsis or septic shock: a nationwide cohort study. PLoS One. 2020;15(12):e0243990. 33373376 10.1371/journal.pone.0243990 PMC7771849

[25] DeFronzo R, Fleming GA, Chen K, et al. Metformin-associated lactic acidosis: current perspectives on causes and risk. Metabolism. 2016;65(2):20–29. 26773926 10.1016/j.metabol.2015.10.014

[26] Buford TW. (Dis)trust your gut: the gut microbiome in age-related inflammation, health, and disease. Microbiome. 2017;5(1):80. 28709450 10.1186/s40168-017-0296-0 PMC5512975

[27] Pryor R, Norvaisas P, Marinos G, et al. Host-microbe-drug-nutrient screen identifies bacterial effectors of metformin therapy. Cell. 2019;178(6):1299–312.e29. 31474368 10.1016/j.cell.2019.08.003 PMC6736778

[28] Karlsson FH, Tremaroli V, Nookaew I, et al. Gut metagenome in European women with normal, impaired and diabetic glucose control. Nature. 2013;498(7452):99–103. 23719380 10.1038/nature12198

[29] Forslund K, Hildebrand F, Nielsen T, et al. Disentangling type 2 diabetes and metformin treatment signatures in the human gut microbiota. Nature. 2015;528(7581):262–266. 26633628 10.1038/nature15766 PMC4681099

[30] Elbere I, Kalnina I, Silamikelis I, et al. Association of metformin administration with gut microbiome dysbiosis in healthy volunteers. PLoS One. 2018;13(9):e0204317. 30261008 10.1371/journal.pone.0204317 PMC6160085

[31] Bryrup T, Thomsen CW, Kern T, et al. Metformin-induced changes of the gut microbiota in healthy young men: results of a non-blinded, one-armed intervention study. Diabetologia. 2019;62(6):1024–1035. 30904939 10.1007/s00125-019-4848-7 PMC6509092

[32] Rhee SH. Lipopolysaccharide: basic biochemistry, intracellular signaling, and physiological impacts in the gut. Intest Res. 2014;12(2):90–95. 25349574 10.5217/ir.2014.12.2.90 PMC4204704

[33] Ahmadi S, Razazan A, Nagpal R, et al. Metformin reduces aging-related leaky gut and improves cognitive function by beneficially modulating gut microbiome/goblet cell/mucin axis. J Gerontol Ser A, Biol Sci Med Sci. 2020;75(7):e9–e21. 32129462 10.1093/gerona/glaa056 PMC7302182

[34] Chen Y, Yu Q, Gong CX. Molecular connection between diabetes and dementia. Adv Exp Med Biol. 2019;1128:103–131. 31062327 10.1007/978-981-13-3540-2_6

[35] Cornick S, Tawiah A, Chadee K. Roles and regulation of the mucus barrier in the gut. Tissue Barriers. 2015;3(1-2):e982426. 25838985 10.4161/21688370.2014.982426 PMC4372027

[36] Mouries J, Brescia P, Silvestri A, et al. Microbiota-driven gut vascular barrier disruption is a prerequisite for non-alcoholic steatohepatitis development. J Hepatol. 2019;71(6):1216–1228. 31419514 10.1016/j.jhep.2019.08.005 PMC6880766

[37] Sun J, Ding X, Liu S, et al. Adipose-derived mesenchymal stem cells attenuate acute lung injury and improve the gut microbiota in septic rats. Stem Cell Res Ther. 2020;11(1):384. 32894198 10.1186/s13287-020-01902-5 PMC7487801

[38] Kong C, Gao R, Yan X, et al. Probiotics improve gut microbiota dysbiosis in obese mice fed a high-fat or high-sucrose diet. Nutrition (Burbank, Los Angeles County, Calif). 2019;60:175–184. 10.1016/j.nut.2018.10.002 30611080

[39] Koh A, De Vadder F, Kovatcheva-Datchary P, et al. From dietary fiber to host physiology: short-chain fatty acids as key bacterial metabolites. Cell. 2016;165(6):1332–1345. 27259147 10.1016/j.cell.2016.05.041

[40] Zhou D, Pan Q, Xin FZ, et al. Sodium butyrate attenuates high-fat diet-induced steatohepatitis in mice by improving gut microbiota and gastrointestinal barrier. World J Gastroenterol. 2017;23(1):60–75. 28104981 10.3748/wjg.v23.i1.60 PMC5221287

[41] Kelly CJ, Zheng L, Campbell EL, et al. Crosstalk between microbiota-derived short-chain fatty acids and intestinal epithelial HIF augments tissue barrier function. Cell Host Microbe. 2015;17(5):662–671. 25865369 10.1016/j.chom.2015.03.005 PMC4433427

[42] Wang K, Lv L, Yan R, et al. Bifidobacterium longum R0175 protects rats against d-galactosamine-induced acute liver failure. mSphere. 2020;5(1). 10.1128/mSphere.00791-19 PMC6992372 31996423

[43] Koh GY, Kane AV, Wu X, et al. Parabacteroides distasonis attenuates tumorigenesis, modulates inflammatory markers and promotes intestinal barrier integrity in azoxymethane-treated A/J mice. Carcinogenesis. 2020;41(7):909–917. 32115637 10.1093/carcin/bgaa018

[44] Nakano H, Wu S, Sakao K, et al. Bilberry anthocyanins ameliorate NAFLD by improving dyslipidemia and gut microbiome dysbiosis. Nutrients. 2020;12(11). 10.3390/nu12113252 PMC7690841 33114130

[45] Koh GY, Kane A, Lee K, et al. Parabacteroides distasonis attenuates toll-like receptor 4 signaling and Akt activation and blocks colon tumor formation in high-fat diet-fed azoxymethane-treated mice. Int J Cancer. 2018;143(7):1797–1805. 29696632 10.1002/ijc.31559

[46] Zhang X, Zhao Y, Xu J, et al. Modulation of gut microbiota by berberine and metformin during the treatment of high-fat diet-induced obesity in rats. Sci Rep. 2015;5:14405. 26396057 10.1038/srep14405 PMC4585776

[47] de la Cuesta-Zuluaga J, Mueller NT, Corrales-Agudelo V, et al. Metformin is associated With higher relative abundance of mucin-degrading akkermansia muciniphila and several short-chain fatty acid-producing microbiota in the gut. Diabetes Care. 2017;40(1):54–62. 27999002 10.2337/dc16-1324

[48] Titov AA, Baker HV. Metformin inhibits the type 1 IFN response in human CD4(+) T cells. J Immunol. 2019;203(2):338–348. 31160534 10.4049/jimmunol.1801651 PMC6615983

[49] Bharath LP, Agrawal M, McCambridge G, et al. Metformin enhances autophagy and normalizes mitochondrial function to alleviate aging-associated inflammation. Cell Metab. 2020;32(1):44–55.e6. 32402267 10.1016/j.cmet.2020.04.015 PMC7217133

[50] Luo YP, Jiang L, Kang K, et al. Hemin inhibits NLRP3 inflammasome activation in sepsis-induced acute lung injury, involving heme oxygenase-1. Int Immunopharmacol. 2014;20(1):24–32. 24583148 10.1016/j.intimp.2014.02.017

[51] Liang H, Ding X, Yu Y, et al. Adipose-derived mesenchymal stem cells ameliorate acute liver injury in rat model of CLP induced-sepsis via sTNFR1. Exp Cell Res. 2019;383(1):111465. 31201811 10.1016/j.yexcr.2019.06.010

[52] Douglas GM, Maffei VJ. PICRUSt2 for prediction of metagenome functions. Nat Biotechnol. 2020;38(6):685–688. 32483366 10.1038/s41587-020-0548-6 PMC7365738

