# Supplementary figures and images for: Metformin attenuated sepsis-related liver injury by modulating gut microbiota
Source: Emerg Microbes Infect. 2022 Mar 15;11(1):815–28. doi: 10.1080/22221751.2022.2045876 (PMC8928825; doi:10.1080/22221751.2022.2045876)

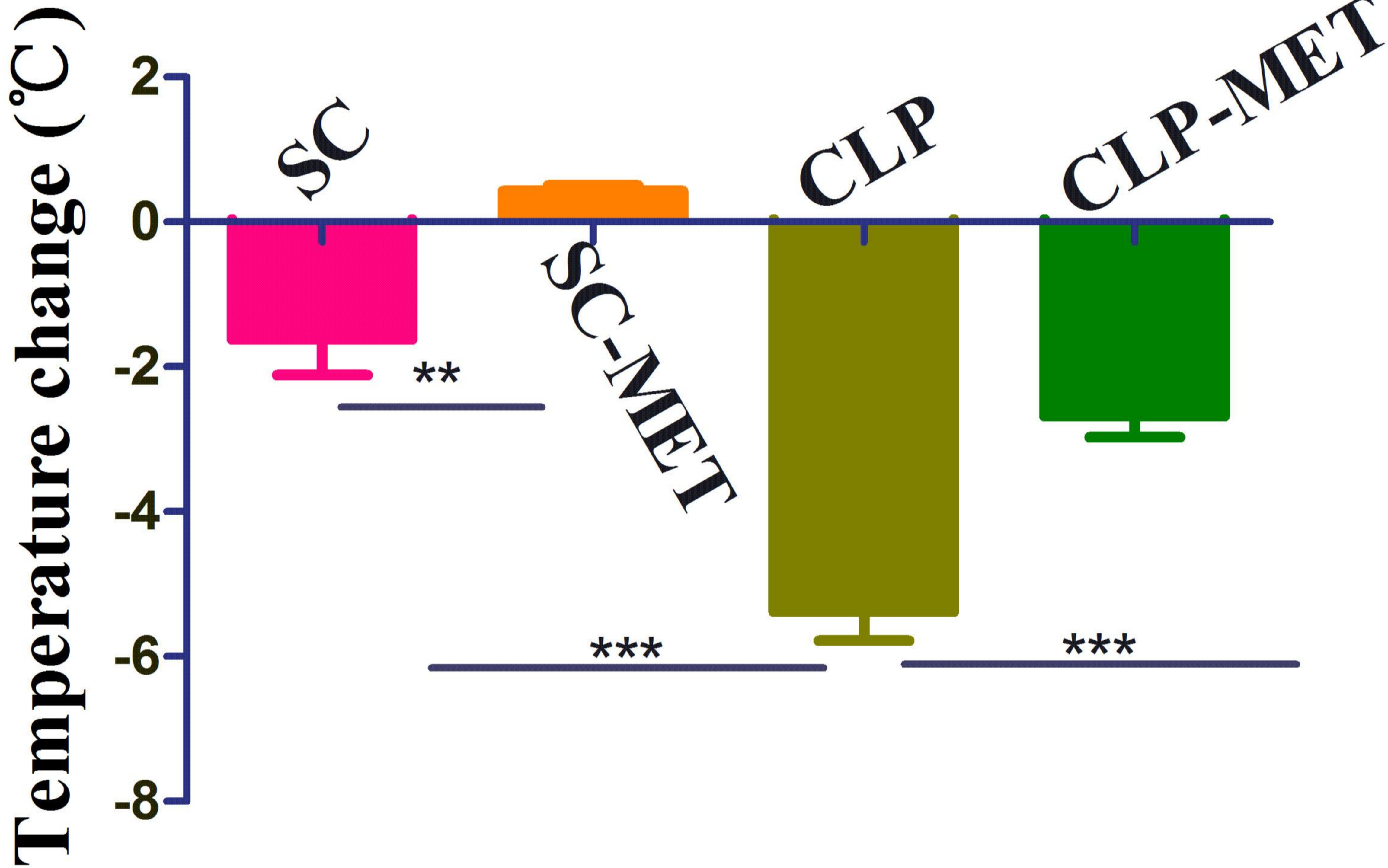

Supplement: Supplemental Material [file TEMI_A_2045876_SM9839.zip › Supplemental figure 1-7/Supplememtal figure 1.pdf]

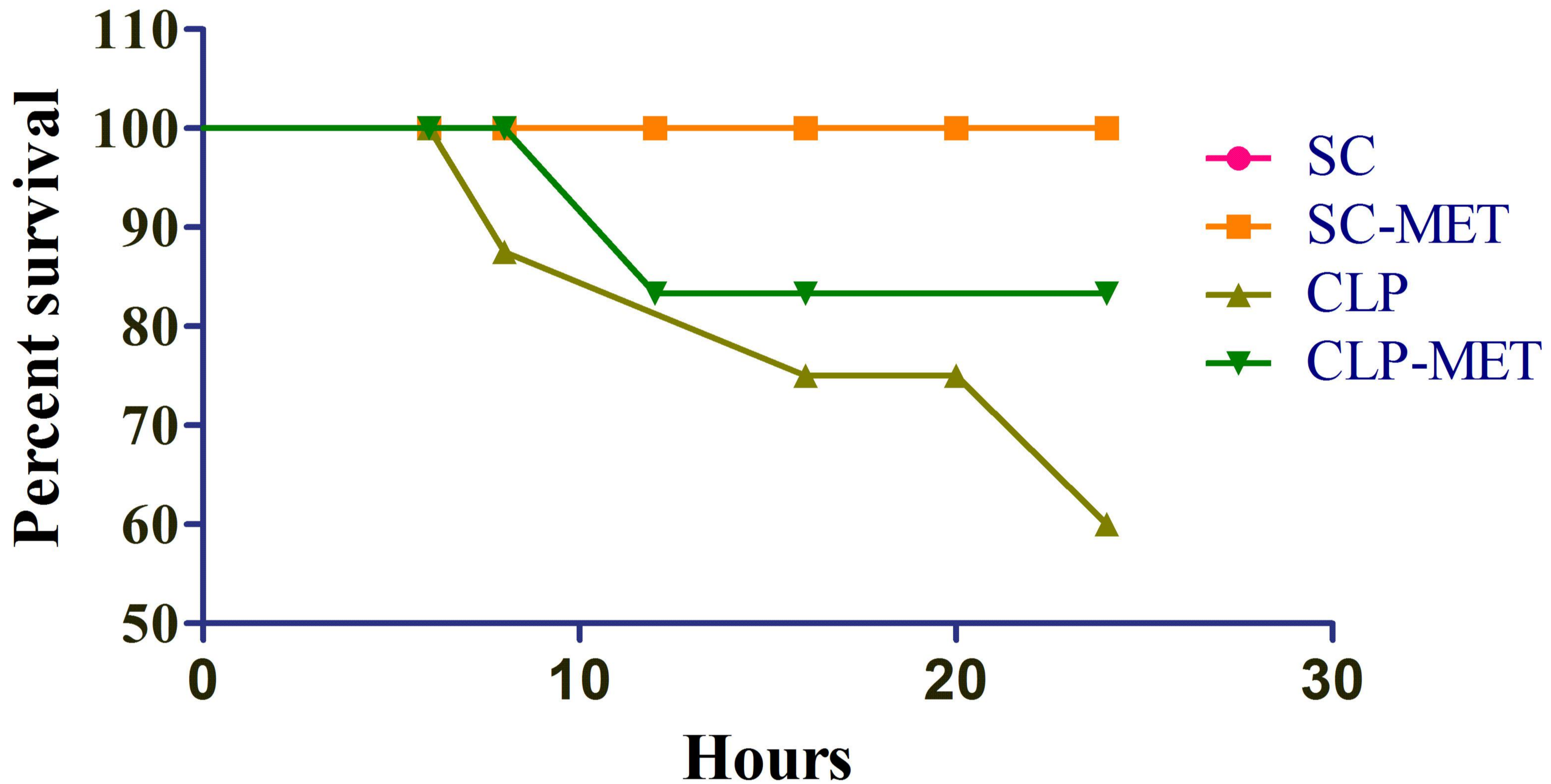

Supplement: Supplemental Material [file TEMI_A_2045876_SM9839.zip › Supplemental figure 1-7/Supplemental figure 2.pdf]

**A**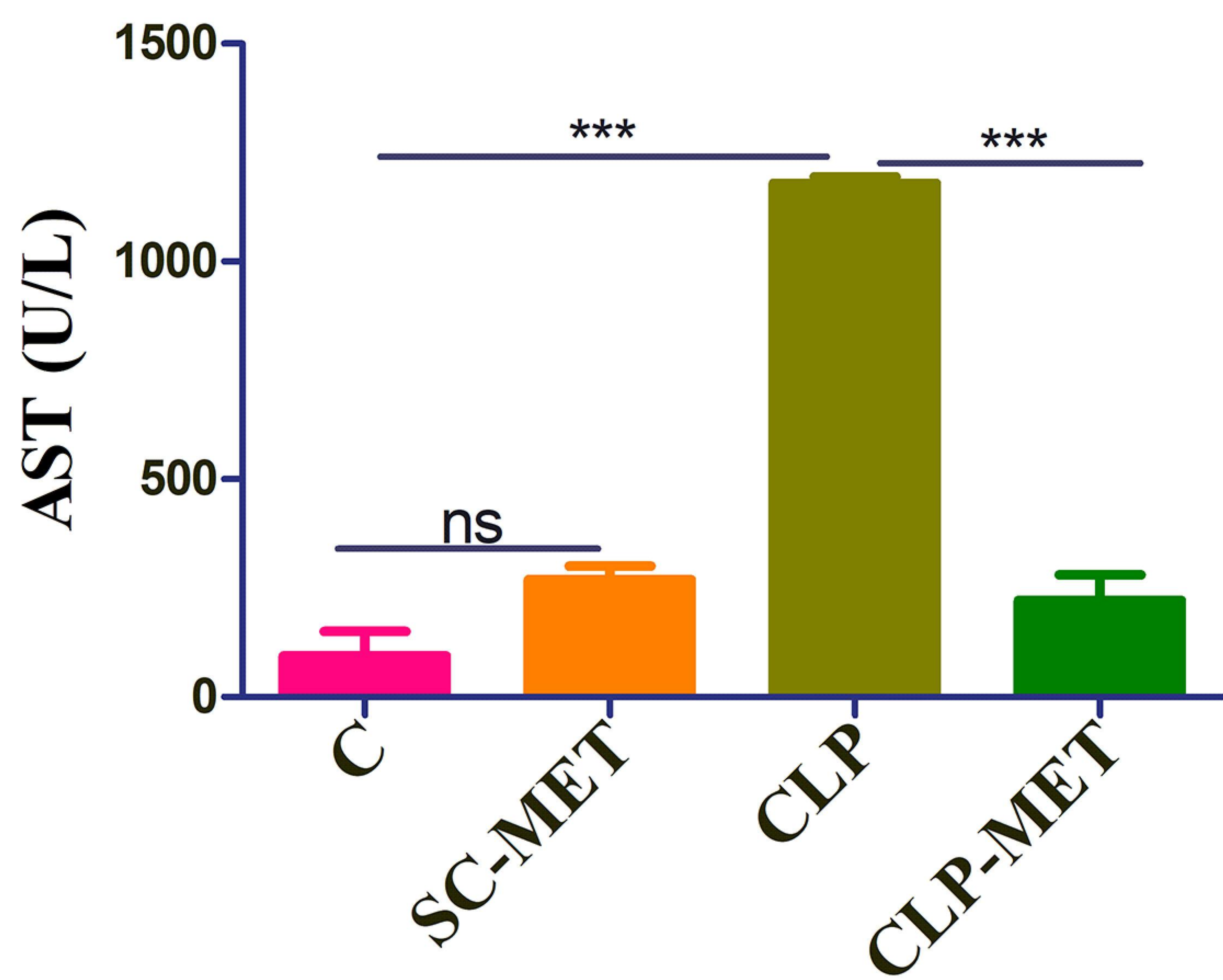**B**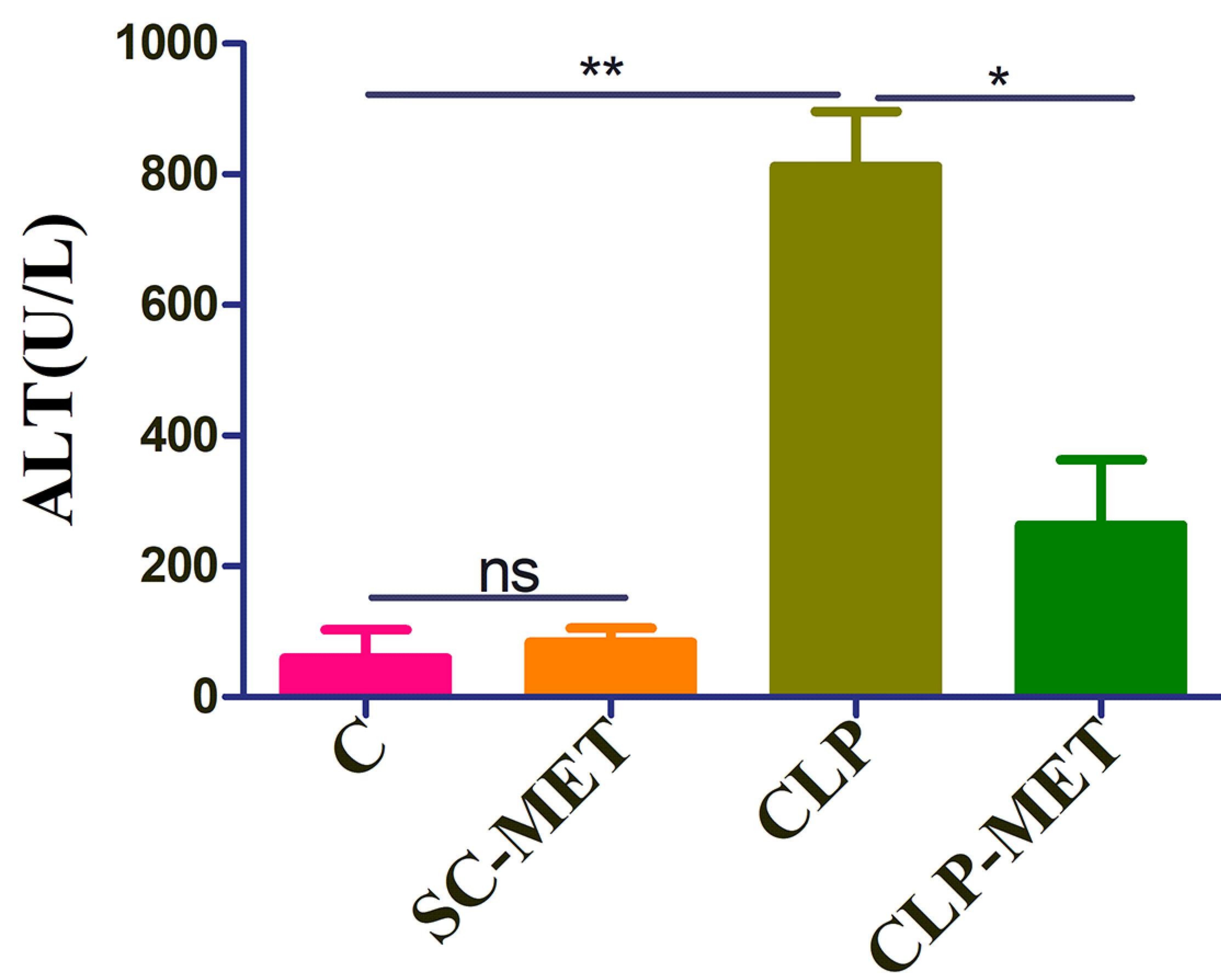**C**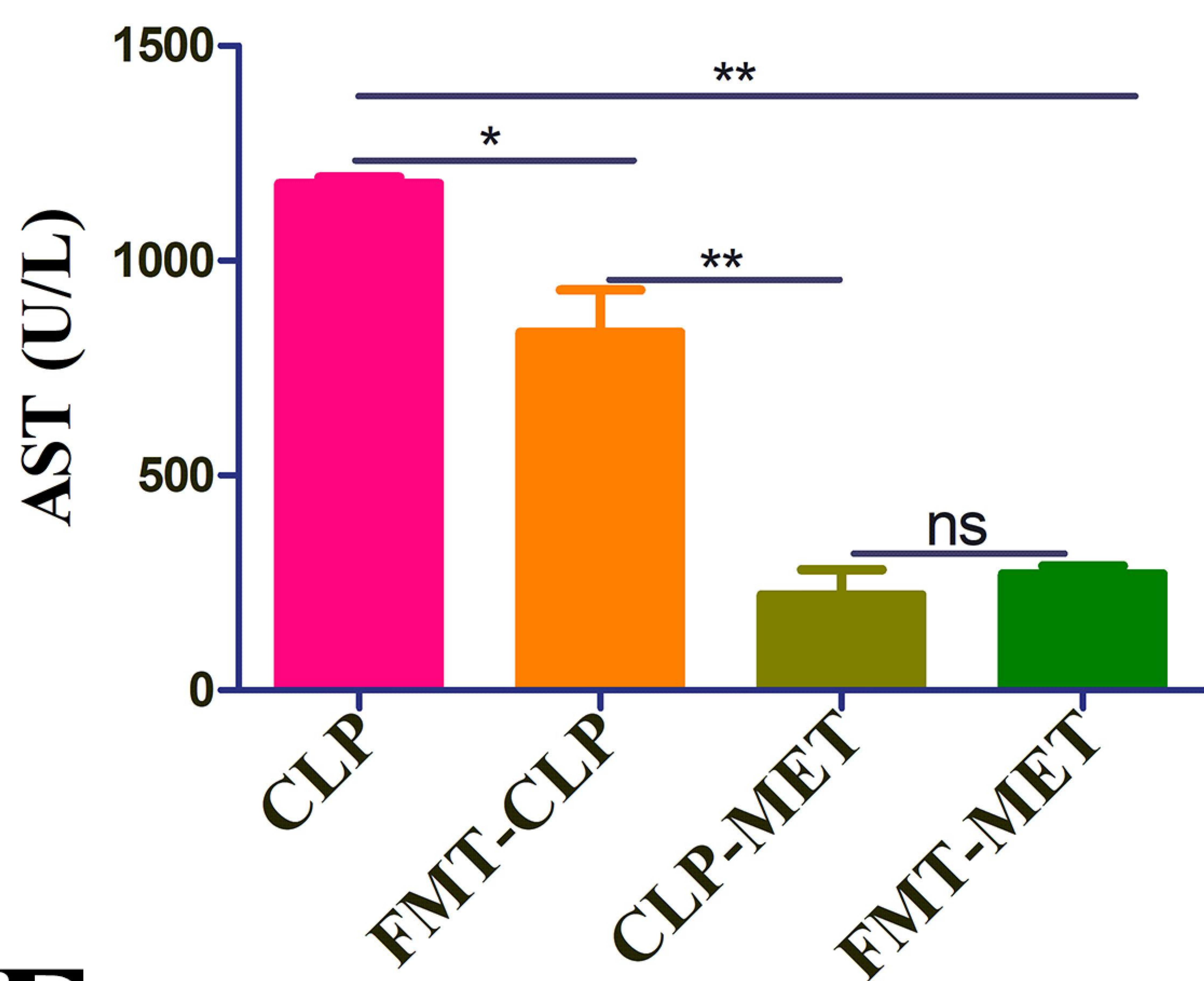**D**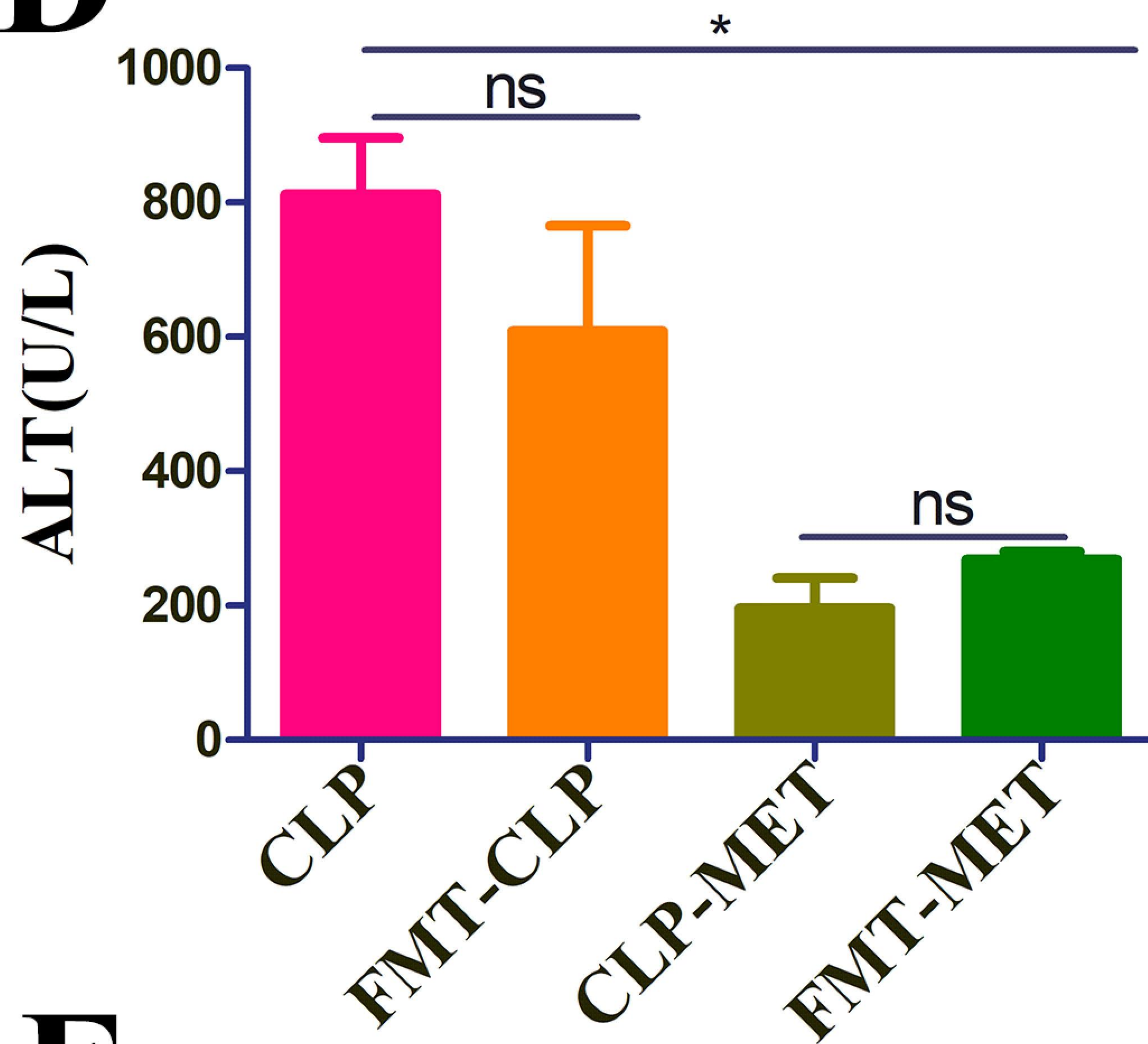**E**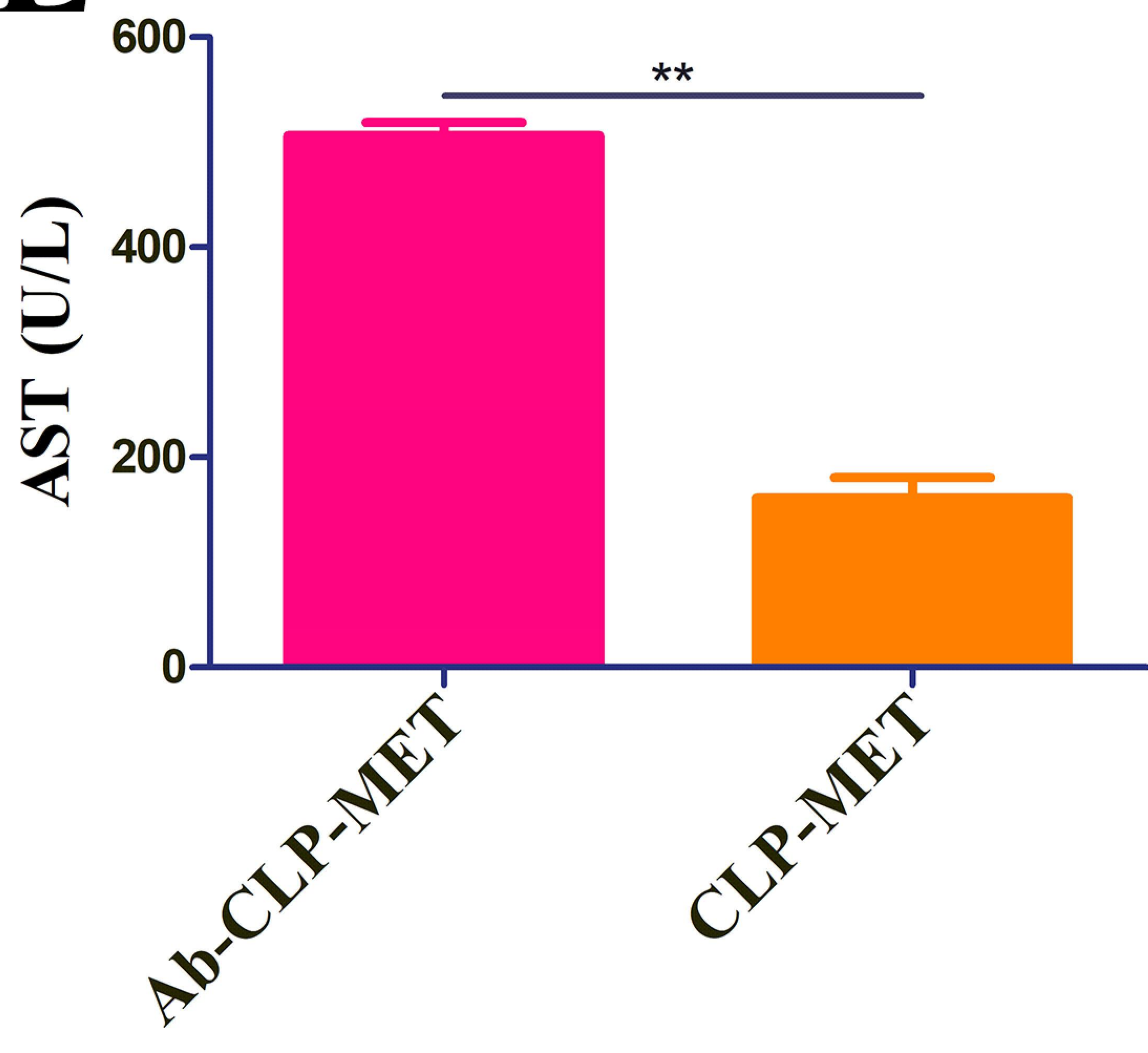**F**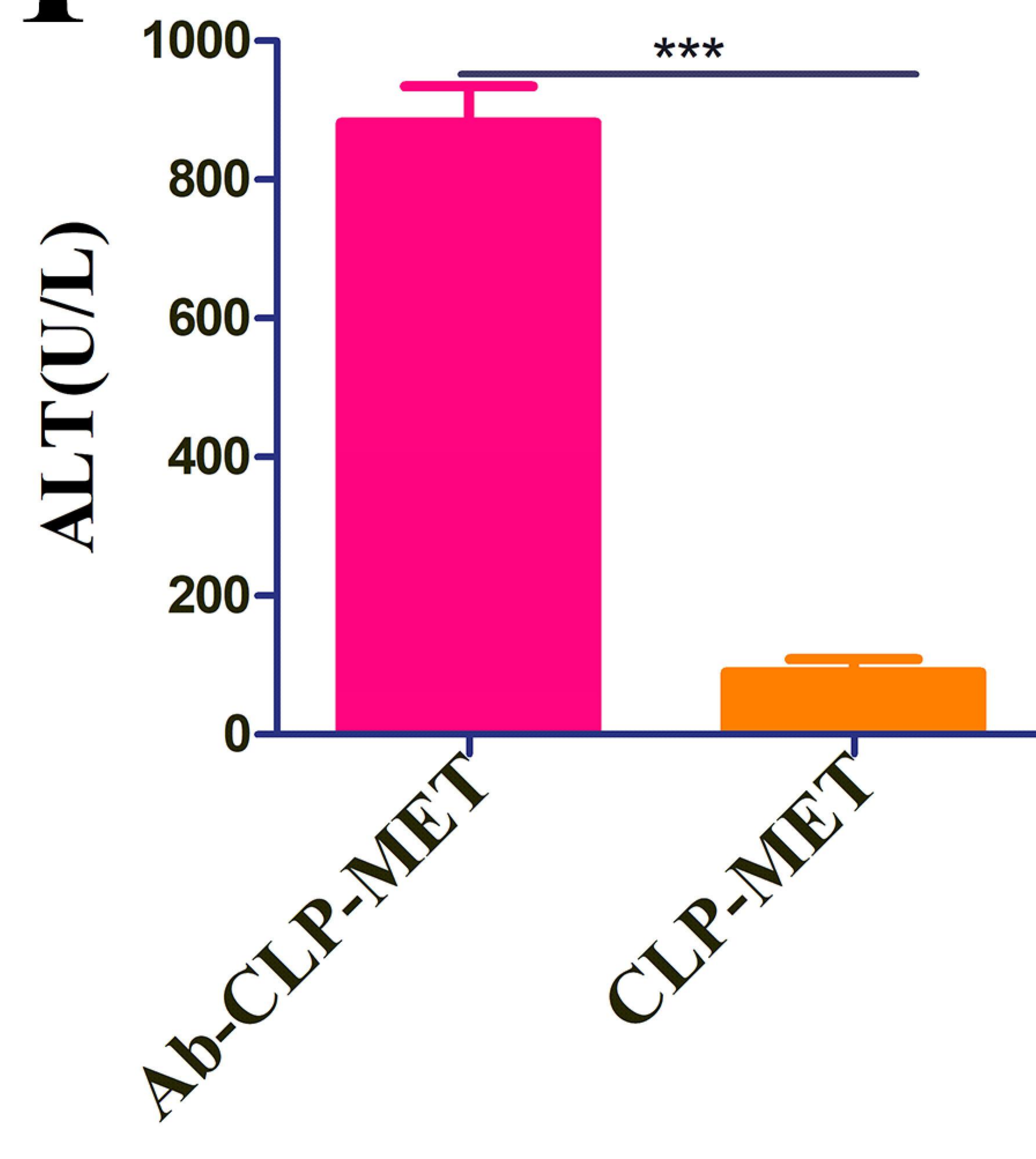

Supplement: Supplemental Material [file TEMI_A_2045876_SM9839.zip › Supplemental figure 1-7/Supplementary Figure 4.pdf]
