# Supplementary material for: Metformin attenuated sepsis-related liver injury by modulating gut microbiota
Source: Emerg Microbes Infect. 2022 Mar 15;11(1):815–28. doi: 10.1080/22221751.2022.2045876 (PMC8928825; doi:10.1080/22221751.2022.2045876)
Supplement: Supplemental Material [file Supplementary Figure 3.pdf › Supplemental figure 1-7/Supplementary Figure 3.pdf]

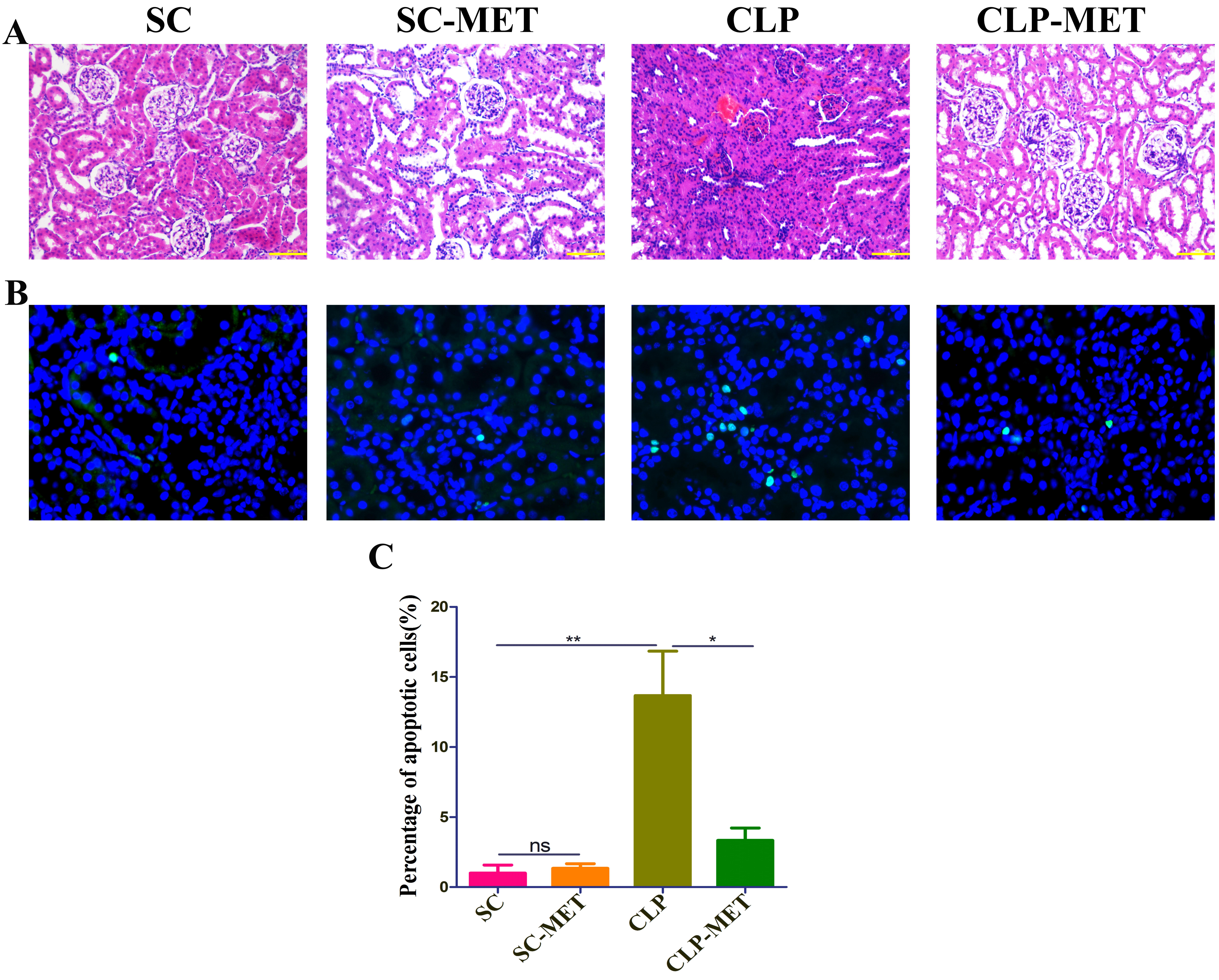
A
B
C
SC
SC-MET
CLP
CLP-MET
Percentage of apoptotic cells(%)
20
15
10
5
0
**
*
ns
SC
SC-MET
CLP
CLP-MET
