# Supplementary material for: Metformin attenuated sepsis-related liver injury by modulating gut microbiota
Source: Emerg Microbes Infect. 2022 Mar 15;11(1):815–28. doi: 10.1080/22221751.2022.2045876 (PMC8928825; doi:10.1080/22221751.2022.2045876)
Supplement: Supplemental Material [file Supplementary Figure 5.pdf › Supplemental figure 1-7/Supplementary Figure 5.pdf]

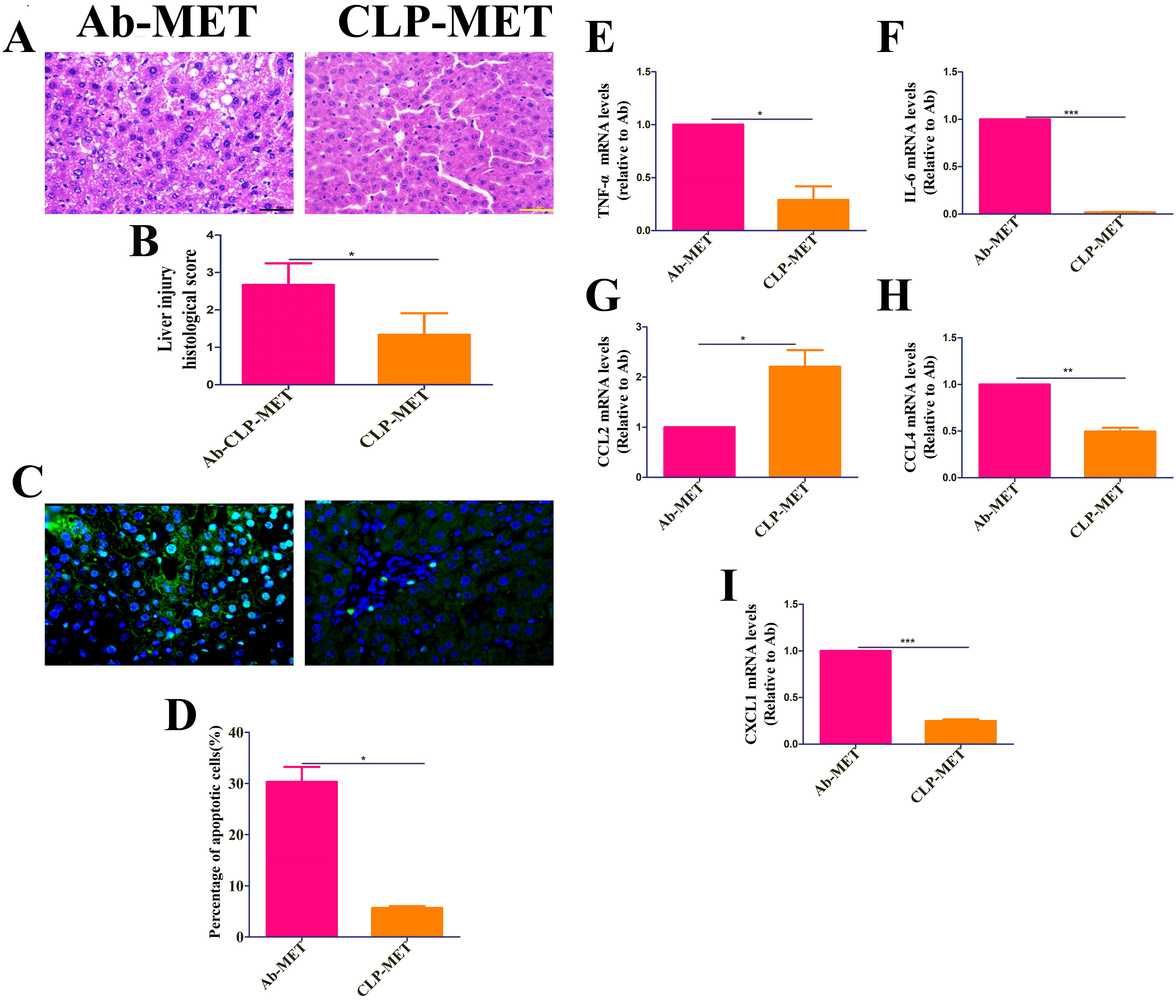

A
Ab-MET
CLP-MET
B
Liver injury histological score
Ab-CLP-MET
CLP-MET
C
D
Percentage of apoptotic cells(%)
Ab-MET
CLP-MET
E
TNF-α mRNA levels (relative to Ab)
F
IL-6 mRNA levels (Relative to Ab)
G
CCL2 mRNA levels (Relative to Ab)
H
CCL4 mRNA levels (Relative to Ab)
I
CXCL1 mRNA levels (Relative to Ab)
*
**
***
