# Supplementary material for: Metformin attenuated sepsis-related liver injury by modulating gut microbiota
Source: Emerg Microbes Infect. 2022 Mar 15;11(1):815–28. doi: 10.1080/22221751.2022.2045876 (PMC8928825; doi:10.1080/22221751.2022.2045876)
Supplement: Supplemental Material [file Supplementary Figure 6.pdf › Supplemental figure 1-7/Supplementary Figure 6.pdf]

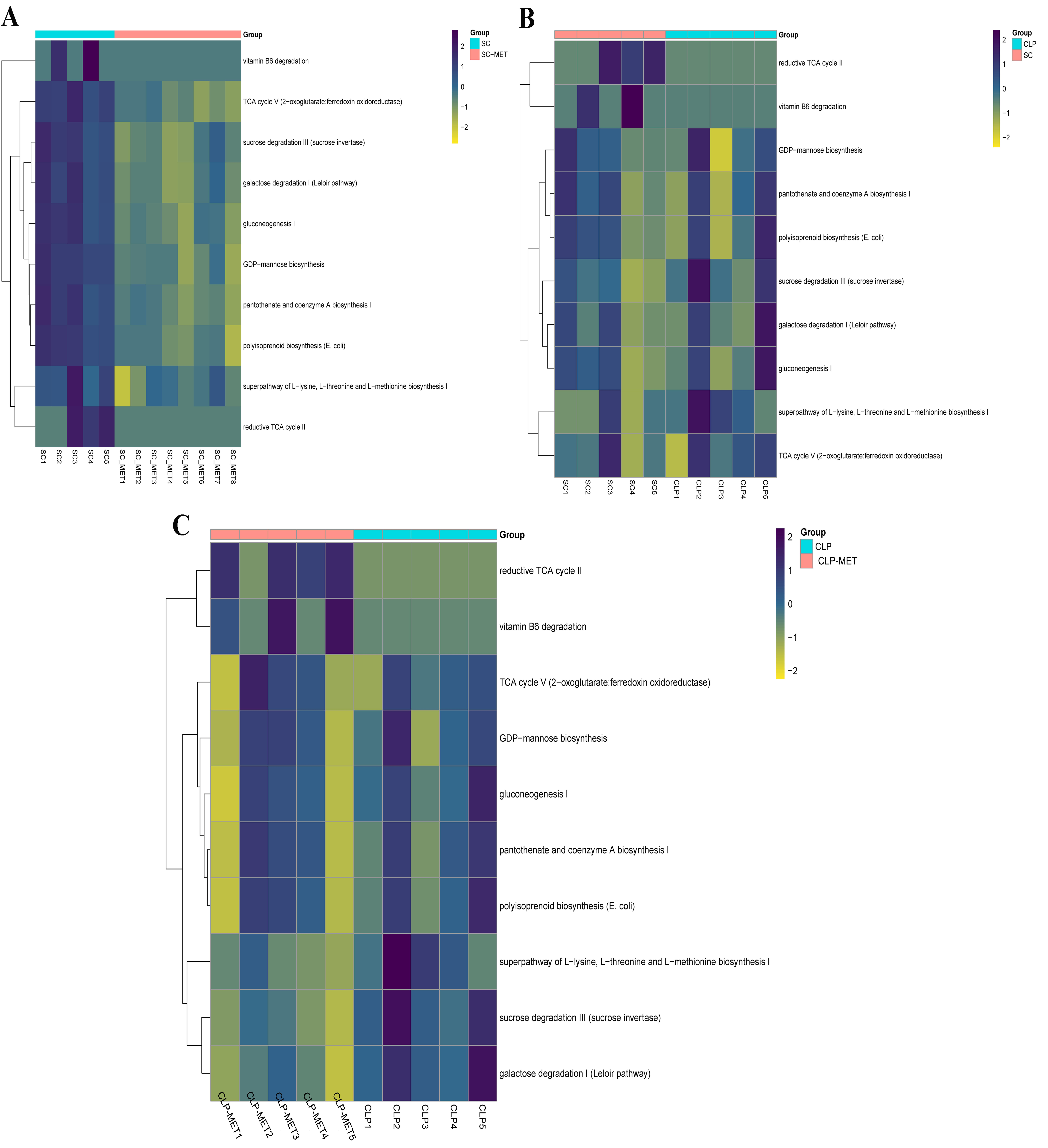
A
Group
SC
SC−MET
2
1
0
−1
−2
vitamin B6 degradation
TCA cycle V (2−oxoglutarate:ferredoxin oxidoreductase)
sucrose degradation III (sucrose invertase)
galactose degradation I (Leloir pathway)
gluconeogenesis I
GDP−mannose biosynthesis
pantothenate and coenzyme A biosynthesis I
polyisoprenoid biosynthesis (E. coli)
superpathway of L−lysine, L−threonine and L−methionine biosynthesis I
reductive TCA cycle II
SC1
SC2
SC3
SC4
SC5
SC_MET1
SC_MET2
SC_MET3
SC_MET4
SC_MET5
SC_MET6
SC_MET7
SC_MET8
B
Group
CLP
SC
2
1
0
−1
−2
reductive TCA cycle II
vitamin B6 degradation
GDP−mannose biosynthesis
pantothenate and coenzyme A biosynthesis I
polyisoprenoid biosynthesis (E. coli)
sucrose degradation III (sucrose invertase)
galactose degradation I (Leloir pathway)
gluconeogenesis I
superpathway of L−lysine, L−threonine and L−methionine biosynthesis I
TCA cycle V (2−oxoglutarate:ferredoxin oxidoreductase)
SC1
SC2
SC3
SC4
SC5
CLP1
CLP2
CLP3
CLP4
CLP5
C
Group
CLP
CLP-MET
2
1
0
−1
−2
reductive TCA cycle II
vitamin B6 degradation
TCA cycle V (2−oxoglutarate:ferredoxin oxidoreductase)
GDP−mannose biosynthesis
gluconeogenesis I
pantothenate and coenzyme A biosynthesis I
polyisoprenoid biosynthesis (E. coli)
superpathway of L−lysine, L−threonine and L−methionine biosynthesis I
sucrose degradation III (sucrose invertase)
galactose degradation I (Leloir pathway)
CLP-MET1
CLP-MET2
CLP-MET3
CLP-MET4
CLP-MET5
CLP1
CLP2
CLP3
CLP4
CLP5
