# Supplementary material for: Metformin attenuated sepsis-related liver injury by modulating gut microbiota
Source: Emerg Microbes Infect. 2022 Mar 15;11(1):815–28. doi: 10.1080/22221751.2022.2045876 (PMC8928825; doi:10.1080/22221751.2022.2045876)
Supplement: Supplemental Material [file Supplementary Figure 7.pdf › Supplemental figure 1-7/Supplementary Figure 7.pdf]

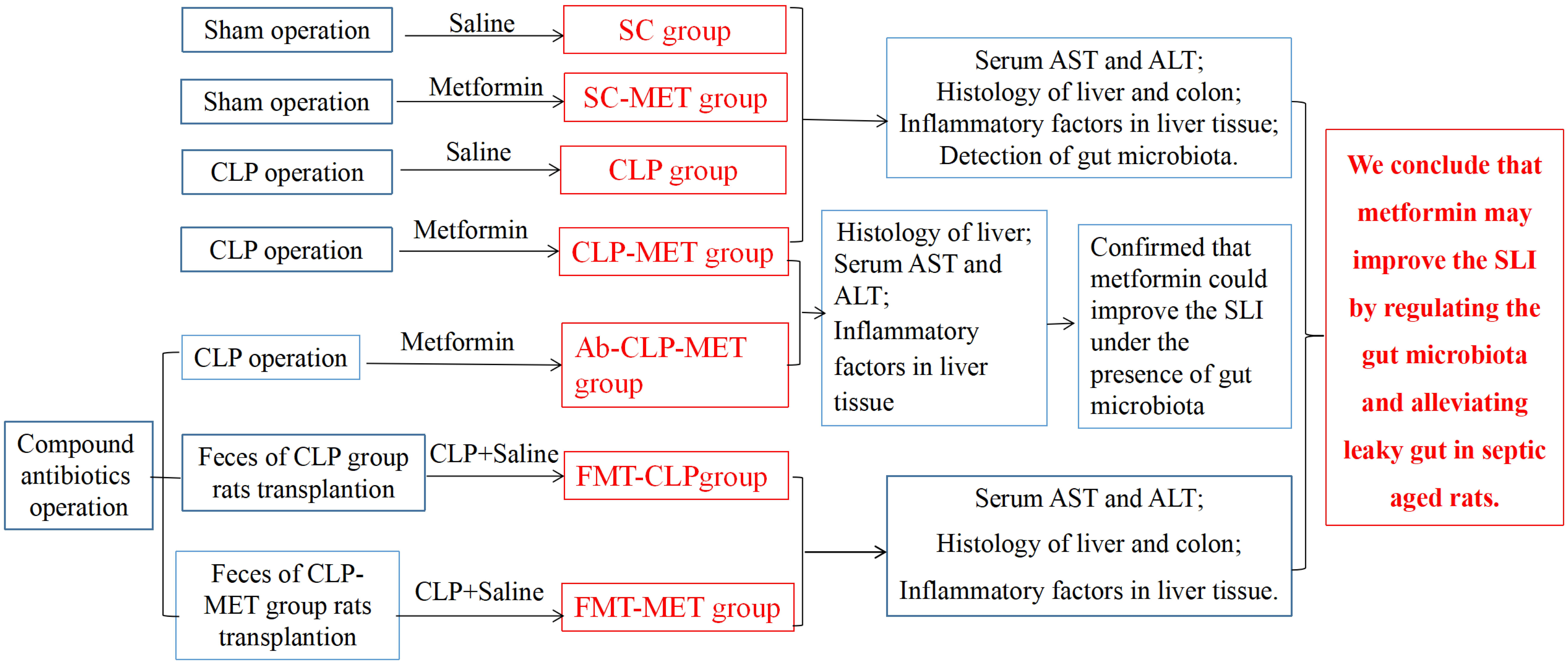

Sham operation
Saline
SC group
Sham operation
Metformin
SC-MET group
CLP operation
Saline
CLP group
CLP operation
Metformin
CLP-MET group
CLP operation
Metformin
Ab-CLP-MET group
Compound antibiotics operation
Feces of CLP group rats transplantion
CLP+Saline
FMT-CLPgroup
Feces of CLP-MET group rats transplantion
CLP+Saline
FMT-MET group
Serum AST and ALT; Histology of liver and colon; Inflammatory factors in liver tissue; Detection of gut microbiota.
Histology of liver; Serum AST and ALT; Inflammatory factors in liver tissue
Confirmed that metformin could improve the SLI under the presence of gut microbiota
Serum AST and ALT; Histology of liver and colon; Inflammatory factors in liver tissue.
We conclude that metformin may improve the SLI by regulating the gut microbiota and alleviating leaky gut in septic aged rats.
